# A top–down neural circuit for affective-motivational responses of pain relief induced by electroacupuncture

**DOI:** 10.1186/s13020-026-01349-5

**Published:** 2026-03-01

**Authors:** Hui Liu, Xin Jia, Zouqin Huang, Yong Xia, Jun Rong, Meiyu Chen, Can Wang, Chuan Qin, Jiaqi Lu, Qiuyong Li, Xueyong Shen, Ling Zhang, Sheng Liu

**Affiliations:** 1 Shanghai University of Traditional Chinese Medicine, Shanghai, 201203 China; 2 Department of Neurology and Neurological Rehabilitation, Shanghai Yangzhi Rehabilitation Hospital, School of Medicine, Tongji University, Shanghai, 201619 China; 3 Shanghai Pudong New Area Hospital of Traditional Chinese Medicine, Shanghai, 201300 China; 4 Hangzhou Red Cross Hospital, Hangzhou, 310003 China; 5 Shiyan Hospital of Traditional Chinese Medicine, Hubei University of Traditional Chinese Medicine, Shiyan, 442002 China; 6 Shanghai Research Center for Acupuncture and Meridians, Shanghai, 201203 China

**Keywords:** Electroacupuncture, Pain, Affective-motivational behaviors, Infralimbic cortex, Accumbens shell, Glutamate

## Abstract

**Background:**

Peripheral neuromodulation, which can be considered as a flow of signals from the body to the brain, influences mental and psychological states. However, whether peripheral neuromodulation, particularly electroacupuncture (EA), may regulate specific neural circuits and evoke affective‒motivational responses remains elusive. Here, we investigate the affective-motivational responses of pain relief following the application of EA in human and animal models in the context of pain.

**Methods:**

The conditioned place preference (CPP), open field test and elevated plus maze tests were used to examine the affective‒motivational responses of pain relief induced by EA in different animal models of pain. EA at acupoint ST36 (2 Hz) was administered. Multi‒electrode array recording, optogenetics, retrograde neuronal tracing, chemogenetics and immunohistochemistry were used to explore the neural circuit mechanisms involved. rAAV virus were used to identify the target projection neurons. A battery of self-report questionnaire was used to assess affective‒motivational responses after EA in patients with chronic low back pain.

**Results:**

EA analgesia induced CPP only in pain states in different animal models of pain. Chronic pain induced negative affective valence of pain. EA attenuated anxious- or depressive-like behaviors in spared nerve injury (SNI) rats. EA robustly activated glutamatergic neurons in the infralimbic cortex (IL) in a pain-dependent manner. The optogenetic activation of IL glutamatergic (IL^Glu^) neurons mimicked EA-induced analgesia and CPP whereas their inhibition reversed the effects promoted by EA. Furthermore, the IL-nucleus accumbens (NAc) shell pathway was activated by EA in SNI rats. Inhibition of IL^Glu^ to the NAc shell reversed EA-induced analgesia, CPP and anxiolytic-like behaviors. In addition, we identified that activation of IL^Glu^ to nucleus accumbens shell^GABA^ projection is necessary for EA induced analgesia, CPP and anxiolytic-like behaviors.

**Conclusions:**

These results illustrate an example in which the emotional dimension of pain is directly influenced through the peripheral neuromodulation and provide the basis for the use of EA to target top down neural circuits to relieve chronic pain in psychological and clinical situations.

***Trial registration*:**

ChiCTR1800020029.

**Supplementary Information:**

The online version contains supplementary material available at 10.1186/s13020-026-01349-5.

## Introduction

Almost 5000 years ago, ancient people discovered the practice of peripherally stimulating specific points of their body to alleviate various diseases or conditions [1]. This technique, known as peripheral neuromodulation, has gained worldwide recognition over the past several decades because of its rapid development and new insights into the diagnosis and treatment of diseases [2]. A growing body of evidence supports the use of acupuncture and electroacupuncture (EA), popular peripheral neuromodulation techniques that target specific points in the body, as effective treatments for acute and chronic pain, including headaches, neck pain, and osteoarthritis pain [3–5]. Research shows that its analgesic mechanisms are partly explained by the gate control theory and involve somatosensory autonomic reflexes, and the peripheral and central opioid, dopamine, noradrenaline, and serotonin systems [6, 7]. While these experimental efforts have largely focused on the perception of pain [8], the various observations made thus far are not sufficient to permit a unified theory regarding the effects of EA.

Current conceptualizations of pain in humans are predominantly multidimensional, including the perception of the noxious stimulus and the affective‒motivational features of pain. Chronic pain can induce maladaptive affective and motivational states, leading to opioid overdose, neuropsychiatric comorbidities and marked impairments in social functioning [2]. Affective and motivational processes not only affect the perception of pain, but also engage physiological mechanisms involved in the descending modulation of nociceptive signals [9]. Furthermore, affective and motivational responses drive patients to seek treatment and can cause them to alter their lifestyle to avoid pain [10]. Notably, recent evidence supports peripheral neuromodulation with acupuncture or EA as more than a tool for the treatment of pain perception [11, 12]. Ramineni et al. reported that EA improved disability, depression and mood in patients with pain [13]. Fang et al. suggested that EA alleviates pain-related anxiety-like behaviors and aversive memory [14, 15]. In our clinical practice, patients often reported that their fatigue, lethargy, and constant tiredness are markedly improved with pain relief after EA treatment, which motivated us to further investigate whether and how peripheral neuromodulation induces emotional changes following the relief of pain. Unveiling the mechanisms by which EA integrates pain affective and motivational states is of particular interest in the context of treating pain and might shed new light on the effects of peripheral neuromodulation in different domains.

Mounting evidence suggests that EA stimulation induces widespread responses in many brain areas, including the prefrontal cortex, the limbic system, and subcortical gray structures, which may provide an important central circuitry basis for integrating pain emotion and shaping behavior via peripheral stimulation with EA [16, 17]. However, the specific findings of these studies have not been entirely consistent, and it is not clear whether observed alterations are a cause or a consequence of pain [3]. The medial prefrontal cortex (mPFC) might be an ideal candidate for top–down control of EA, especially given its role in processing complex stimulus‒response information and the modulation of sensory and affective components of pain [18]. Importantly, it has been shown that the mPFC is deactivated in chronic pain conditions and, conversely, that its activation results in analgesic effects and may provide important control for the aversive response to transient noxious stimulation [19, 20]. Studies suggest that peripheral neuromodulation with EA involves mPFC modulation [21]. For example, EA analgesia was potentiated in association with neurostimulation of the infralimbic (IL) subregion of the mPFC. Inactivation of synaptic transmission in the IL by local microinjection of cobalt chloride blocked EA-induced analgesia [22]. Work from our laboratory has shown that EA promotes an increase in the bursting activity of IL neurons in rats, indicating that acupuncture can modulate the activity of IL neurons [16, 23]. However, the precise cell type-specific organization and function of cortical circuits that mediate EA-induced analgesia remain elusive. In this study, we specified that the IL glutamatergic (IL^Glu^) to nucleus accumbens (NAc) shell gamma-aminobutyric acid (GABA)-ergic (NAc shell^GABA^) pathway is involved in EA-induced analgesia, and illustrated an example by which the affective-motivational dimension of pain is directly influenced through top–down control of the peripheral neuromodulation with EA.

## Methods

### Animals

Adult male Sprague–Dawley rats (250–350 g; Slack) were purchased from the Shanghai Laboratory Animal Center at the Chinese Academy of Science (Shanghai, China). Male C57BL/6J mice (6–8 weeks old), purchased from the SLAC laboratory (Shanghai), and CamKIIα-Cre (6–8 weeks old), a gift from Zhang Yuqiu's laboratory at Fudan University, were used for the experiments. All the animals were raised under stable conditions maintained at a controlled temperature of 23 ± 3 °C and an air humidity of 40–70% under a 12-h light/dark cycle (lights on from 7:00 A.M. to 7:00 P.M.) with ad libitum access to food and water. Behavioral tests were performed in the dark, and the animals were allowed to adapt to the environment for 1 week before the experiment was performed. All experiments were approved by the Institutional Animal Care and Use Committee and the Animal Ethics Committee (SZY 201710008) of Shanghai University of Traditional Chinese Medicine. The experiments were conducted at the Laboratory of Experimental Acupuncture, Shanghai University of Traditional Chinese Medicine, and Key Laboratory of Spine and Spinal Cord Injury Repair and Regeneration of the Ministry of Education, Tongji University, in accordance with local guidelines for animal welfare.

### Animal models of spared nerve injury (SNI)

The rats were anesthetized with isoflurane (5%). To separate the biceps femoris and to expose the sciatic nerve and its associated branches, we made an incision proximal to the lateral side of the right knee and thereafter separated the biceps femoris. Then, we exposed the sciatic nerve and its branches. We used a silk suture to ligate the common peroneal and tibial nerve branches. Approximately 1 mm of the nerve was removed, and the sural nerve was kept intact. A sham operation was performed as a control, following the same surgical procedure, but the sciatic nerve, peroneal nerve, and tibial nerve branches were not cut off or ligated.

### Animal models of incisional injury pain (INP)

Injury to the skin plus deep tissue, including the fascia and underlying muscle, was performed as described by Woo et al. [24]. The rats were anesthetized with 2% (vol/vol) isoflurane, a 1-cm longitudinal incision was made through the skin of the left hind paw, and the plantaris muscle was elevated and incised longitudinally. The cut skin was stitched with two 5–0 nylon sutures, and the wound site was treated with systemic antiseptic solution (benzylpenicillin sodium, 60,000 units). Sham-operated animals were anesthetized, and the left hind paw was removed, but no incision was made.

### Animal models of monosodium iodoacetate injection (MIA)

MIA was dissolved in sterile saline at a concentration of 3 mg/50 μL. The rats were anesthetized with a 2% isoflurane O2 mixture and given a single intraarticular injection of 3 mg MIA through the patellar ligament on the left. The animals in the control group were given a single intraarticular injection of 50 μL of sterile saline. To induce significant spontaneous pain (manifested as obvious limping), the conditioning processes were started 24 h after injection.

### EA treatment

The rats were restrained gently and subjected to EA with two stainless steel needles (0.16 mm diameter, 13 mm length; Suzhou Yuwell Group, China) inserted bilaterally into the ST36, SP6, BL23 or nonacupuncture points at a depth of 5 mm. Acupuncture point ST36 is located near the knee joint, between the muscle anterior tibialis and the muscle extensor digitorum longus. BL23 is located at one to two rib widths lateral to the caudal border of the spinous process of the second lumbar vertebra. SP6 is located 3 mm proximal to the superior border of the medial malleolus. The nonacupuncture point is located 1/5 the length of the tail from the proximal region of the tail (one needle). The other needle was inserted into the anterior tibial muscle 3 mm distal to ST36. This site is a commonly used non-acupoint control in rodent EA studies, located away from known meridians and major nerve trunks [46]. Constant current square wave electric stimulation produced by using a stimilator (Model G-6805-2, Shanghai Medical Electronic Apparatus, China) was transferred via the needles. The stimulation frequency was 2 Hz. The intensity of the stimulation was 2 mA for 30 min. As a sham control, the needles were inserted into ST36, but electric stimulation was not conducted. For the mice, acupuncture needles 0.16 mm in diameter were inserted at a depth of 2–3 mm into the bilateral ST36. The parameters were set as follows: 2 Hz frequency and 2 mA intensity for a total of 30 min. For the sham EA treatment, acupuncture needles were inserted into the mice without electrical stimulation.

To test the cumulative effects of EA, the rats were subjected to EA every other day from day 7–42 after SNI and were measured on days 14, 28, 35 and 42. For anxious- or depressive-like behaviors (EPM, OFT, SPT, and FST), the animals were subjected to EA every other day from day 19 to day 28 after SNI.

### Nociceptive behavior

To examine thermal pain, we used an IITC Model 390 Paw Stimulator Analgesia Meter (IITC/Life Science Instruments, United States) to measure the paw withdrawal latency (PWL). We placed the rats or mice in an inverted plastic cage. After the rats or mice were acclimatized for 30 min, radiant heat was used to warm the hind paws of the rats or mice until their paws were lifted. We adjusted the radiant heat intensity to induce a response at approximately 12–13 s in normal rats or mice. Thereafter, the PWL scores were analyzed by calculating the time from the onset of radiant heat application to paw withdrawal. The test was repeated three times independently for each animal to calculate the mean values of the PWL scores. In addition, the magnitude of hyperesthesia was also evaluated with the difference score (DS), which was calculated by subtracting the PWL of the sham-operated paw from the PWL of the operated paw.

To test mechanical sensitivity, the mice were placed in a plexiglass box with a grid at the bottom and adjusted for at least 30 min before allodynia measurement. A series of Von Frey filaments (0.16 g as a starting dose, RWD Life Science Co., Ltd., China) were applied to stimulate the lateral one-third of the right paws (in the distribution of the sural nerve) of the mice vertically according to the method of “up and down” for measuring mechanical allodynia. For the rats, von Frey filaments were applied to the lateral third of the right paw, and a 50% withdrawal threshold was calculated.

### Conditional place preference (CPP)

CPP was performed as described previously [25]. A standard two-chamber balanced design was used, with EA/light conditioning sessions occurring for 3 days (rats) or 4 days (mice). On the basis of our preliminary findings, 3 days of EA conditioning in rats and 4 days of EA conditioning in mice can induce reliable and stable CPP. Behavior tracking of the animals was recorded by a camera (JLBehv CCD, Jiliang Technology Co., Ltd. China) and analyzed via a behavior analysis system (ANY-maze video tracking software, Stoelting Co. USA). Two conditioning sessions (EA/light stimulation and gentle handling) were performed. The animals were subjected to either EA/light stimulation or gentle handling for 30 min each in the morning and afternoon. We immediately placed the animals in the pairing chamber and confined them to the chamber for 30 min after EA/light stimulation or gentle handling. For every conditioned animal, EA/light stimulation and gentle handling were carried out alternatively in the morning and afternoon. The morning and afternoon EA/light stimulation and gentle handling were conducted at least 4 h apart. A preference test was conducted after conditioning. The animals were placed in the CPP apparatus with a door opened in the middle for 15 min to allow access to the apparatus’s whole parts. The preference scores were analyzed by calculating the amount of time the animals spent in the EA/light stimulation paired chamber minus the time they spent in the gentle handling chamber. The sham EA group was subjected to sham EA.

### Elevated plus maze (EPM)

An EPM apparatus (Shanghai Jiliang Technology Co. Ltd. China) was used for this study. This maze was made of two open arms facing in opposite directions and two closed arms facing in opposite directions that were 50 cm above the floor (all arms were 15 cm wide and 45 cm in diameter). As described previously [26], the rats were placed in the center of the maze facing an open arm at the beginning of the test and were allowed to roam freely for 15 min, during which time a video tracking apparatus was used to record their movement and behavior with ANY-maze video tracking software (Stoelting Co. USA). Both the distance in the open arms and the total distance were measured as exploratory behaviors of interest, with an arm entry being recorded when rats or mice entered a given maze arm with all four paws.

### Open field test (OFT)

OFT analyses are routinely used when anxiety and spontaneous locomotor activity are measured. For rats, the central zone was defined as a 50 × 50 cm area in the center of the 100 × 100 cm open field apparatus. For mice, the central zone was defined as a 20 × 20 cm area in the center of the 40 × 40 cm open field apparatus. The rats or mice were then allowed to freely move through this apparatus for 15 min, during which time a video-tracking system (ANY-maze video tracking software, Stoelting Co. USA) automatically recorded and analyzed the distance in the central area and the total distance covered by these animals.

### Sucrose preference test (SPT)

The animals were acclimated to the test room for at least 20 min before the test. Two bottles (1% sucrose solution vs. tap water) were presented to the rats individually for 24 h. Each rat had free access to both a 1% sucrose solution bottle and a tap water bottle for 24 h. Sucrose and tap water intakes were measured by volume before and after the test. The total liquid consumption was the sum of sucrose water and tap water consumption. The sucrose preference of each rat was expressed as the percentage of sucrose water consumption divided by the total water consumption.

### Forced swimming test (FST)

The animals were placed in a polystyrene cylinder (20 cm high and 15 cm in diameter) containing water (16 cm deep, 24–25°C). The animals were videotaped with a video-tracking system (Shanghai XinRuan Information Technology Co. Ltd.) for 6 min. Immobility was defined as a lack of swimming and minimal movement of one leg. The duration of immobility within the last 4 min of the test was scored manually in a double-blinded manner.

### Immunofluorescence staining

The rats or mice were deeply anesthetized via pentobarbital sodium (100 mg/kg, intraperitoneal (i.p.), sequentially perfused transcardially with saline (200 mL for rats, 30 mL for mice) and 4% paraformaldehyde (PFA) in 0.1 mol/L phosphate buffer (PB) (250 mL for rats, 30 mL for mice), and the brains were collected from each animal, fixed overnight in 4% PFA, and transferred to 30% sucrose for 5‒7 days. The samples were then cut to yield a series of 30 μm-thick coronal sections. The staining of these sections for c-Fos was conducted as described in our prior study [25]. Briefly, the sections were washed three times with PBS, blocked for 2 h with 1% BSA at 4 °C, and probed for 48 h with primary antibodies, including anti-c-Fos (1:500; #ab208942, Abcam, UK), anti-vGlut2 (1:400; #42-7800, Thermo Fisher Scientific, USA), anti-CamKII (1:1000; #ab52476, Abcam, UK) and anti-GABA (1:1000; #A2052, Sigma, USA), at 4 °C. Following three subsequent washes with PBS, the sections were stained for 2 h with the corresponding fluorescent secondary antibody (1:500; Sigma, USA). A Leica laser scanning confocal microscope was then used to analyze these stained sections. An automatically generated 250 × 600 μm rectangle was used to denote the IL and the NAc shell in each section, and analytical software (ImageJ, NIH, USA) was used to calculate the number of stained nuclei per section. The number of positive nuclei per section was thereby determined and averaged to yield representative results for analysis.

### Subcellular fractionation and western blotting

The animals were anesthetized with 2% isoflurane and decapitated immediately. We removed the brains of the animals quickly and collected tissue from the NAc shell on ice. Synaptoneurosome fractions were prepared as described previously [27]. NAc shell samples were homogenized in ice-cold solution A (1 mM MgCl2, 1 mM NaHCO3, 0.1 mM phenylmethane sulfonyl fluoride, 0.32 M sucrose, 0.5 mM CaCl2, and 1× complete protease inhibitors (Thermo Fisher Scientific, USA)). The homogenates were subsequently centrifuged at 4000 rpm for 10 min, after which the supernatants were collected. The pellet was rehomogenized in solution A and centrifuged again at 3000 rpm for 10 min. The combined supernatants were subjected to a second centrifugation at 3000 rpm for 10 min. The supernatants were then spun at 14,000 rpm for 30 min. The pellet was resuspended in solution B (0.32 M sucrose, 1 mM NaHCO3) and homogenized. The homogenate was layered on top of a 5 mL 1 M sucrose and 1.2 M sucrose gradient and centrifuged at 30,000 rpm for 2 h. Purified synaptosomes were collected at the 1 M and 1.2 M sucrose interfaces, suspended in solution B, and centrifuged at 40,000 rpm for 45 min. Synaptosomal pellets were resuspended in 25 mM Tris with 4% SDS. The following antibodies were used: anti-GluA1 (1:1000; #YT1923; Immunoway, USA), anti-GluA2 (1:1000; #YT1921; Immunoway, USA), and anti-GAPDH (1:2000; #ZC-12001; Zcibio, China).

### Extracellular recording

Animals (weighing 300–350 g prior to surgery) were anesthetized with an intraperitoneal injection of sodium pentobarbital (induction, 50 mg/kg; maintenance, 15 mg/kg) and placed in a stereotaxic device. Sixteen stainless steel microwires (50 μm diameter) were arranged in the array and implanted in the IL (3.0 mm AP, 0.6 mm ML, 5.0 mm DV from the bregma) of the rats. The rats were reared in a single cage and injected with penicillin (160 000 IU/day) until 5 days after the operation. Neurophysiological recordings were performed before and after EA stimulation. The animals were connected to the recording apparatus (Cerebus, Blackrock Neurotech, USA), which consisted of a headstage attached to operational amplifiers, a cable, and a commutator to allow nonrestriction to the animals’ movement. The signals were amplified 500 × via a multichannel amplifier. The spikes were filtered between 100 Hz and 8 kHz. Neuronal activity was sampled at 40 kHz, high-pass filtered at 250 Hz, sorted online and stored via the Cerebus acquisition system. Cross-correlograms were examined to prevent analysis of the same unit recorded on different channels. The raw data were preprocessed via Offline Sorter V2.8 (Cerebus, Blackrock Neurotech, USA). The raw signals were low-cut filtered (250 Hz) before waveform detection. Clusters (unites) associated with individual neurons were isolated automatically via K-means and valley-seeking algorithms following principal component analysis. We divided the IL neurons into putative glutamatergic pyramidal cells and interneurons on the basis of their firing rates and spike widths [28]. One cluster contained a majority of neurons with a low firing rate (< 15 Hz) and broad spike waveform (> 225 ms; putative excitatory pyramidal neurons), whereas the other contained neurons with high firing rates (> 15 Hz) and narrow spike waveforms (< 225 ms; putative inhibitory interneurons). Putative interneurons were excluded from further analysis because of their low proportion.

Single-unit neuronal activity in the IL was further analyzed via NeuroExplorer (version 5.3) and MATLAB (2017b). The baseline data were extracted for 100 s, and the post-EA stimulation data were extracted for 100 s. Each unit’s activity in the baseline and post-EA stimulation periods was Z score normalized to the mean and the SD of the baseline firing rate. Differences in the baseline firing rate between groups were assessed with the Wilcoxon signed-rank test. The response type of each unit was determined with a Wilcoxon signed-rank test to compare the Z values of neuronal activity before and after EA stimulation. Only those with a statistically significant increase (*P* < 0.05) following EA stimulation were defined as activated. The activities of light-activated neurons in the IL were further compared between the periods within the unpaired and paired chambers. The firing rates inside each chamber were extracted in a 1000 ms bin and a smoothing window of 7 bins. The values were Z-normalized with the means and standard deviations of those in the unconditioned chamber. The group-level difference was determined with a Wilcoxon signed-rank test.

### Intracranial microinjection

The mice were anesthetized with 1% sodium pentobarbital through an intraperitoneal injection of 50 mg/kg sodium pentobarbital and then fixed on a stereotaxic apparatus. The skulls of the mice were exposed through a median incision, and a microinjection glass pipette was used to locate the target brain area according to the mouse or rat atlas of Paxinos and Watson.

For optogenetic stimulation, rAAV-Ef1α-DIO-hChR2-(H134R)-EYFP-WPRE-pA (2.0 × 1012 vg/ml, BrainVTA Co. Ltd., China) was injected into the right IL (relative to bregma: AP, 1.6 mm; ML, − 0.25 mm; DV, − 2.1 mm), or rAAV-Ef1α-DIO-eNpHR3.0-EYFP-WPRE-pA (3.56 × 1012 vg/mL, OBiO Technology Corp. Ltd., China) was injected into the bilateral IL (relative to bregma: AP, 1.6 mm; ML, ± 0.25 mm; DV, − 2.1 mm) at a volume of 80 nL with 20 nL/min in CamKIIα-Cre mice, and rAAV-hEf1α-DIO-EYFP-WPRE-pA (0.48 × 1012 vg/mL) was used as a control. To observe the projection of the IL to the NAc shell, 120 nL of a 1:3 volume mixture of AAV2/1-hSyn-Cre (1.37 × 1012 vg/mL) and rAAV-EF1a-DIO-mcherry-WPRE-pA (1.0 × 1012 vg/mL) was injected into the left IL (relative to the bregma: AP, 1.6 mm; ML, 0.25 mm; DV, − 2.1 mm), and 120 nL of rAAV-hEF1a-DIO-EYFP-WPRE-pA (0.48 × 1012 vg/mL) was injected into the left NAc shell (relative to the bregma: AP, 1.1 mm; ML, 1 mm; DV, − 4.25 mm) in C57BL/6J mice at 20 nL/min. To manipulate the projection of glutamatergic neurons in the IL to the NAc shell, rAAV-Ef1α-DIO-hChR2-(H134R)-EYFP-WPRE-pA (2.0 × 1012 vg/mL) was injected into the bilateral IL (relative to bregma: AP, 1.6 mm; ML, ± 0.25 mm; DV, − 2.1 mm), and rAAV-hEf1α-DIO-EYFP-WPRE-pA (0.48 × 1012 vg/mL) was used as a control. For the chemogenetic experiment, 120 nL of rAAV-EF1a-DIO-hM4D(Gi)-EGFP-WPREs (5 × 1012 vg/mL) or rAAV-EF1a-DIO-EGFP-WPRE-hGH polyA (5 × 1012 vg/mL) was bilaterally injected into the IL (relative to bregma: AP, 1.6 mm; ML, ± 0.25 mm; DV, − 2.1 mm). A total of 120 nl of rAAV-CaMKlla-CRE-WPRE-hGH PA was bilaterally injected into the NAc shell (relative to bregma: AP, 1.1 mm; ML, ± 1 mm; DV, − 4.25 mm). These viruses were generated by BrainVTA Co. Ltd., China. Clozapine oxide (CNO, BrainVTA Co. Ltd.) was dissolved in saline to a concentration of 1.0 mg/mL. CNO (1 mg/kg, i.p.) was injected 30 min before EA stimulation. In our pilot study, it was sufficient to produce hM4Di-mediated inhibition, as indicated by reduced IL-Fos expression in response to EA stimulation (data not shown).

For microinjection of a mixture of M/B into the IL or NBQX into the NAc shell, the rats were intraperitoneally injected with pentobarbital sodium (50 mg/kg) and mounted on a stereotaxic frame, with isoflurane (1.5–2%) being delivered via a nosecone. Bilateral guide cannulae (26 gauge, Plastics One) were implanted in the IL (relative to bregma: 3.0 mm AP; ± 0.6 mm ML; − 4.0 mm DV) or in the NAc shell (relative to bregma: AP, 1.7 mm; ML, ± 0.8 mm; DV, − 6.5 mm). The cannulae were fixed to the skull with dental cement and three steel screws. The rats were systemically treated with benzylpenicillin sodium (60,000 U) to prevent infection. After surgery, the rats were allowed to recover for 7 days. Five minutes prior to EA stimulation, the rats were bilaterally injected with a combination of M/B (0.06 and 0.6 nmol, respectively, Tocris Bioscience, USA) or NBQX (0.55 nmol, Tocris Bioscience, USA) into the IL or the NAc shell. The injection cannulae were inserted into the guide cannulae and extended 1 mm beyond the guide tip. The injectors were attached to a microinfusion pump (RS Instruments, India) via PE 10 tubing. A total of 0.5 µL/side of drug or control was injected into the brain. The injection cannulae were left in place for an additional 2 min to allow diffusion.

### Optic fiber implantation (for the fiber photometry and optogenetic experiments)

Following the viral injection, an optic fiber (diameter, 200 μm; numerical aperture (NA), 0.37, Shanghai June Biotechnology Co., Ltd.) was immediately implanted into the upper right IL (relative to bregma: AP, 1.6 mm; ML, − 0.25 mm; DV, − 2.1 mm). To optogenetically inhibit glutamatergic neurons in the IL during EA stimulation, after the bilateral IL was injected with the virus, the optic fibers were implanted bilaterally above the IL at an angle of 20 degrees (relative to the bregma: AP, 1.6 mm; ML, ± 1.01 mm; DV, − 2.03 mm). To manipulate the projection of glutamatergic neurons in the IL to the NAc shell, after the IL was injected with the virus, the fibers were implanted bilaterally above the NAc shell at an angle of 10 degrees (relative to bregma: AP, 1.1 mm; ML, ± 1.75 mm; DV, − 4.22 mm). Then, gel (Vetbond Adhesive, 3 M) was applied to the skull surface, and dental cement was used to cover the exposed skull and fix the optical fibers. The animals were allowed to recover from anesthesia on an electric blanket before being returned to their home cage. Subsequent experiments were performed 3 weeks after virus expression.

### Optrode implantation for extracellular recording

An optrode consisting of a 200 μm optical fiber coupled to a 16-channel microelectrode array (50 μm tungsten wires) was used for simultaneous optogenetic stimulation and extracellular recording. The mice were anesthetized with 1% sodium pentobarbital through an intraperitoneal injection of 50 mg/kg sodium pentobarbital and then fixed on a stereotaxic apparatus. The skull of each mouse was exposed through a median incision, and three cranial screws were drilled after the skull was drilled. A microinjection glass pipette was used to locate the right IL according to the mouse atlas of Paxinos and Watson. A skull drill was used to drill a window in the IL, and the dura and pia mater were removed. After the virus was injected into the right IL (relative to bregma: AP, 1.6 mm; ML, − 0.25 mm; DV, − 2.1 mm), the electrode was slowly and vertically implanted in the IL. The two ground wires were welded to the two cranial screws. Then, gel (Vetbond Adhesive, 3 M) was applied to the skull surface, and dental cement was used to cover the exposed skull and fix the optrode. The animals were allowed to recover from anesthesia on an electric blanket before being returned to their home cage. Subsequent experiments were performed 3 weeks after virus expression.

### Optogenetic manipulations

The implantable optic fibers were connected to a laser generator via optic fiber sleeves. The delivery of blue light or yellow light was controlled by a Doric system (Doric Lenses, Canada) for 30 min. For optogenetic stimulation, blue light (473 nm, 6 mW, 10 ms pulse duration, 10 Hz train) was delivered for 30 min during conditioning. For inhibition, constant yellow light (589 nm, 5–8 mW) was applied. The same stimulus pattern was applied in the control group. Optogenetic manipulations were performed in the conditioning phase of the CPP paradigm.

### Retrograde tracing

The rats were anesthetized by administering pentobarbital sodium (50 mg/kg, i.p.), and a small hole was drilled through the skull above the NAc shell to remove the dura. A 30-gauge needle was lowered into the right NAc shell (relative to bregma: AP, 1.7 mm; ML, − 0.8 mm; DV, − 6.5 mm). A total of 30 nL of CTb was delivered via pressure injection. Thereafter, the injection was performed over 1 min. After the injection, the needle was left in place for 2 min. CTb injections that diffused outside the NAc shell were not included in this study. The rats were allowed to recover for 7 days after surgery before CPP conditioning and testing. To confirm the potential CTb sites in the NAc shell, the tissue sections were reacted with rabbit anti-CTb antibodies and then incubated for 2 h with Alexa Fluor 488-conjugated anti-rabbit antibodies.

CTb/c-Fos double-label immunohistochemistry was performed via the same procedure as described for c-Fos/vGlut2 double labeling. We used a rabbit c-Fos primary antibody (1:500, #SAB2100833, Sigma, USA) and a goat anti-CTb primary antibody (1:500, #C730, Sigma, USA) as the two primary antibodies. The donkey anti-goat Alexa Fluor 488 (1:500, #SAB4600387, Sigma, USA) and goat anti-rabbit Alexa Fluor 647 (1:500, #AF647, Sigma, USA) antibodies were used as secondary antibodies.

### Clinical observation

Human experimentation was approved by the ethical committee of the Center of Zhejiang Integrated Traditional and Western Medicine Hospital (ITWMH, No. 202101) and was registered at www.chictr.org.cn (Identifier: ChiCTR1800020029). Details regarding the methods are provided in the Supplemental methods.

### Statistical analysis

Statistical analysis was performed using GraphPad Prism 8 and MATLAB. The data were analyzed using unpaired t tests, paired t tests, one-way ANOVA, and two-way ANOVA. *P* < 0.05 was the cutoff of significance for these analyses. The statistical analyses are described in the corresponding figure legends.

## Results

### Peripheral neuromodulation with EA induces analgesia and affective-motivational behaviors

**Fig. 1:**
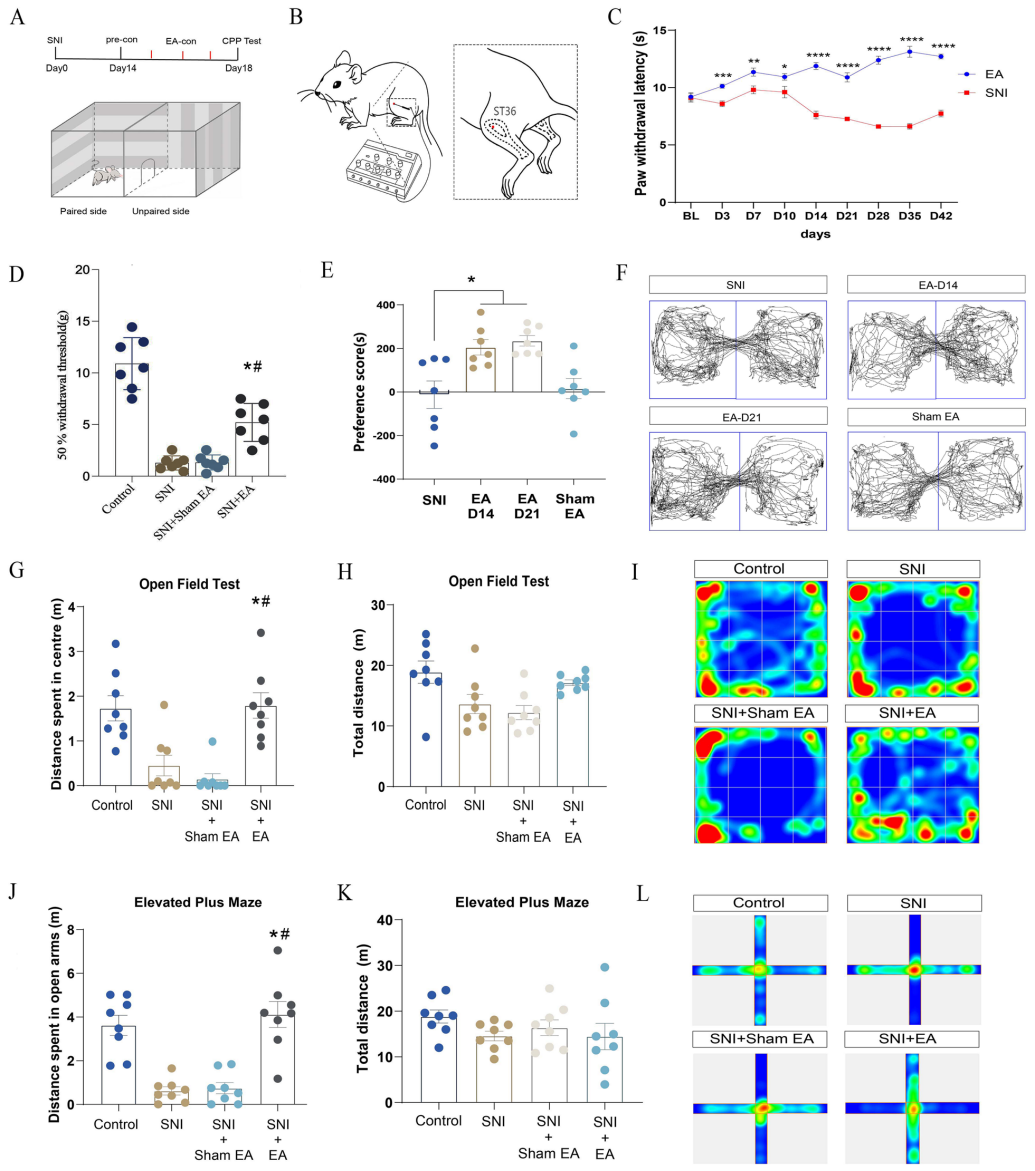
EA induced analgesia and affective‒motivational behaviors in rats with spared nerve injury (SNI). **A** Schematic showing the experimental protocol. **B** Schematic representation of the placement of EA stimulation at ST36. **C** Thermal paw withdrawal latency (F (1, 15) = 16.820, two-way ANOVA with Tukey’s multiple comparisons test, *P* < 0.0001; n = 8–9 rats/group). **D** 50% mechanical withdrawal threshold (g) (F (3,24) = 53.663, n = 7 rats/group). *P < 0.01, SNI + EA versus SNI groups; #*P* < 0.01, SNI + EA versus SNI + Sham EA groups. **E** Preference scores (F (3, 24) = 8.205, *P* < 0.006; n = 7 rats/group). EA-D14 group (preconditioned on day 14 after SNI; EA-conditioned from day 15 to day 17 after SNI). EA-D21 group (preconditioned on day 21 after SNI; EA-conditioned from day 22 to day 24 after SNI). **F** Real-time movement traces during the CPP test. **G** Distance in the central area during the OFT on day 28 after SNI. (F (3, 28) = 12.81, *P* < 0.0001; n = 8 rats/group), *P < 0.05, SNI + EA versus SNI groups; ^#^*P* < 0.01, SNI + EA versus SNI + Sham EA groups. **H** Total distance traveled during the OFT (F (3, 28) = 1.327, *P* > 0.05). **I** Heatmaps for behavior tracking of rats in the four groups during the OFT. **J** Distance traveled in the open arms during the EPM test on day 31 after SNI (F (3, 28) = 20.65, *P* < 0.0001; n = 8 rats/group). *P < 0.05, SNI + EA versus SNI groups; #*P* < 0.05, SNI + EA versus SNI + Sham EA groups. **K** Total distance traveled during the EPM test (F (3, 28) = 1.173, *P* > 0.05). **L** Heatmaps for behavior tracking during the EPM test. All the data in the dot plots are shown as the means ± S.E.M.s. *P < 0.05, **P < 0.01, ***P < 0.001, ****P < 0.0001. One-way ANOVA (**D, E, G, H, J, K**) with Tukey’s multiple comparisons test

We first investigated whether EA induced positive motivational behaviors in different animal models of pain. CPP, which is based on the affective and motivational qualities of pain relief, provides a reliable paradigm for testing the motivational drive to prefer a context associated with pain relief (Fig. 1A) [9, 29]. Preclinical studies have demonstrated that the relief of pain induced by analgesic agents promotes CPP, irrespective of the animal model used [30]. We therefore tested whether the analgesic effect of a well-established EA protocol evoked CPP. We chose 2 Hz EA bilaterally at point ST36 (a depth of 2 mm near the knee joint, between the tibialis anterior and extensor digitorum longus muscles, Fig. 1B) according to the previous studies [31, 32]. In Sprague‒Dawley rats with spared nerve injury (SNI), the thresholds of mechanical allodynia and thermal hyperalgesia were significantly decreased after SNI modeling (Fig. 1C, D). Paw withdrawal latency (PWL) scores were increased after EA (Fig. 1D). The cumulative effects of EA-induced analgesia were maintained for at least 42 days after SNI (Fig. 1C). In the CPP paradigm, one chamber becomes associated with EA through three-day repeated pairings, whereas the other chamber is associated with no EA stimulation (Fig. 1A). Preference scores were measured by taking into consideration the amount of time animals spent in the EA associated chamber minus the time they spent in the non-EA chamber, when given free access to both chambers after conditioning. EA produced a significant preference for the chamber paired with EA that was not found in a group of animals given a sham EA (Fig. 1E, F), suggesting that EA induced CPP in animals with chronic neuropathic pain. In C57BL/6J mice with SNI, EA also relieved mechanical allodynia and thermal hyperalgesia and induced CPP (Figure S1), corroborating previously obtained results.

We next made an incision in the right hind paw of the rats to mimic acute pain. In this incisional injury pain (INP) model, incision-evoked pain hypersensitivity was prominent at 24 h and still present at 96 h after incision (Figure S2), which is consistent with previous studies [30]. EA at the ST36 point, but not at the nonacupuncture points, considerably attenuated pain hypersensitivity in the following 24 h and 48 h postincision (Figure S2B). EA resulted in a strong preference for the chamber paired with EA in INP rats. Interestingly, EA performed at nonacupuncture points in INP rats did not promote pain relief or induce CPP (Figure S2C–D). Notably, EA associated with context in sham-operated animals did not induce CPP, indicating that CPP was only induced by EA when pain was present. Short-term use of EA does not necessarily have intrinsic motivational effects on sham-operated rats. In addition, we observed that EA at acupoint ST36 also induced CPP in a rat model of monoiodoacetate (MIA)-induced arthritis (Figure S3). Taken together, these results demonstrated that EA analgesia can induce CPP in different animal models of pain.

Some acupuncture points are discrete locations of the body, where manual or electrical stimulation can evoke similar analgesic effects. To determine the generality of this preference effect, we further tested whether two other acupuncture points, BL23 (located on the back) and SP6 (located on the leg), which are commonly used for analgesia clinically, could also induce CPP. Indeed, both EA at BL23 and SP6 produced a significant preference for the pair of EA compartments in INP rats (Figure S4).

Chronic pain can induce a negative affective valence of pain, which is associated with opioid overdose and many neuropsychiatric comorbidities. We further investigated whether EA attenuates anxious- or depressive-like behaviors in SNI animals. We found that rats subjected to SNI for 28 days developed anxious or depressive-like behavior (Fig. 1G–L, Figure S5). Accordingly, in the open field test (OFT), SNI rats exhibited a significant reduction in the distance traveled in the center zone, with EA stimulation significantly increasing this parameter during the OFT (Fig. 1G–I). Similarly, EA also significantly increased the number of open arms in the elevated plus maze (EPM) test (Fig. 1J–L). Notably, EA stimulation did not impact total distance traveled during the OFT or EPM test (Fig. 1H, K), indicating that EA did not cause any generalized behavioral impairments. Forced swimming (FST) and sucrose preference (SPT) tests were performed on day 28 and day 31 after SNI (Figure S5A). Consistent with other reports [11, 33], EA significantly shortened the immobility time in the FST (Figure S5B). EA also evoked a tendency toward increased sucrose intake, although this effect did not achieve statistical significance (Figure S5C). These results suggest that EA attenuates pain-related negative emotions.

### EA activates glutamatergic neurons in the IL cortex in rats with pain

**Fig. 2:**
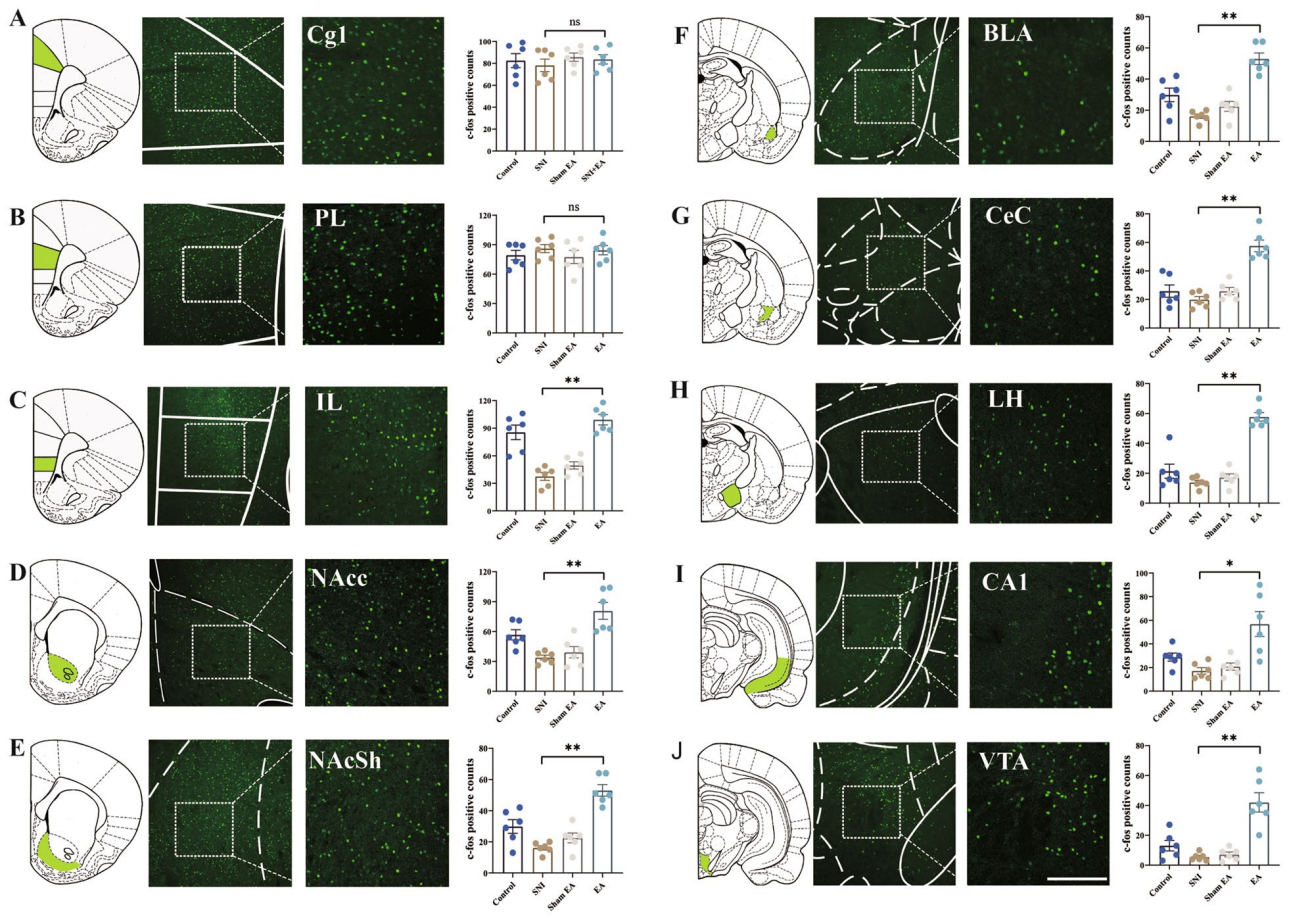
Brain-wide c-fos mapping of regions associated with the affective and motivational aspects of pain induced by EA. Schematic maps showing c-Fos expression in specific regions following EA corresponding to atlas-defined brain regions (left). Magnified regions that are shown within boxes in the middle columns (right). Scale bar, 100 μm. Cg1, area 1 of the cingulate cortex; PL, prelimbic cortex; IL, infralimbic cortex; NAcc, core accumbens; NAcSh, shell accumbens; BLA, basolateral amygdaloid nucleus; CeC, central amygdaloid nucleus; LH, lateral hypothalamus; VTA, ventral tegmental area. All the data are expressed as the means ± S.E.M.s. *P < 0.05; **P < 0.01; ns, not significantly different (*P* > 0.05). n = 6 rats/group. One-way ANOVA with Tukey’s multiple comparisons test was used

We first investigated neuronal activation in the brain associated with affective and motivational aspects of pain [34] after EA stimulation using immediate early gene c-Fos expression mapping in SNI rats. The structures associated with increased EA-induced c-Fos expression included the IL, the NAc shell and core, the basolateral amygdala (BLA), the central nucleus (CeA), the lateral hypothalamus (LH), the hippocampal CA1, and the ventral tegmental area (VTA) (Fig. 2). Notably, EA stimulation increased the number of c-Fos positive neurons in the IL but not in the cingulate (Cg1) or prelimbic (PL) subregions of the mPFC. Sham EA did not increase c-Fos expression in the IL in SNI rats. The results indicate that the relationship between EA and neuronal activation in the mPFC occurs primarily in the IL area of mPFC subregions. In addition, the IL region plays a central role in regulating both goal-directed behaviors and conditioned responses [35, 36]. We reasoned that the IL becomes activated by cues associated with EA-induced pain relief. To test this hypothesis, four or five rats per group were randomly selected for c-Fos immunohistochemistry 90 min after the EA CPP test (Figure S6A). As expected, EA-induced CPP promoted an increase in the number of c-Fos positive neurons in the IL (Figure S6B–H). Interestingly, EA in sham-operated animals, sham EA, and EA at nonacupuncture points did not increase c-Fos expression in the IL. These results indicate that the IL region is also involved in the EA-related cue response in rat models of pain.

**Fig. 3:**
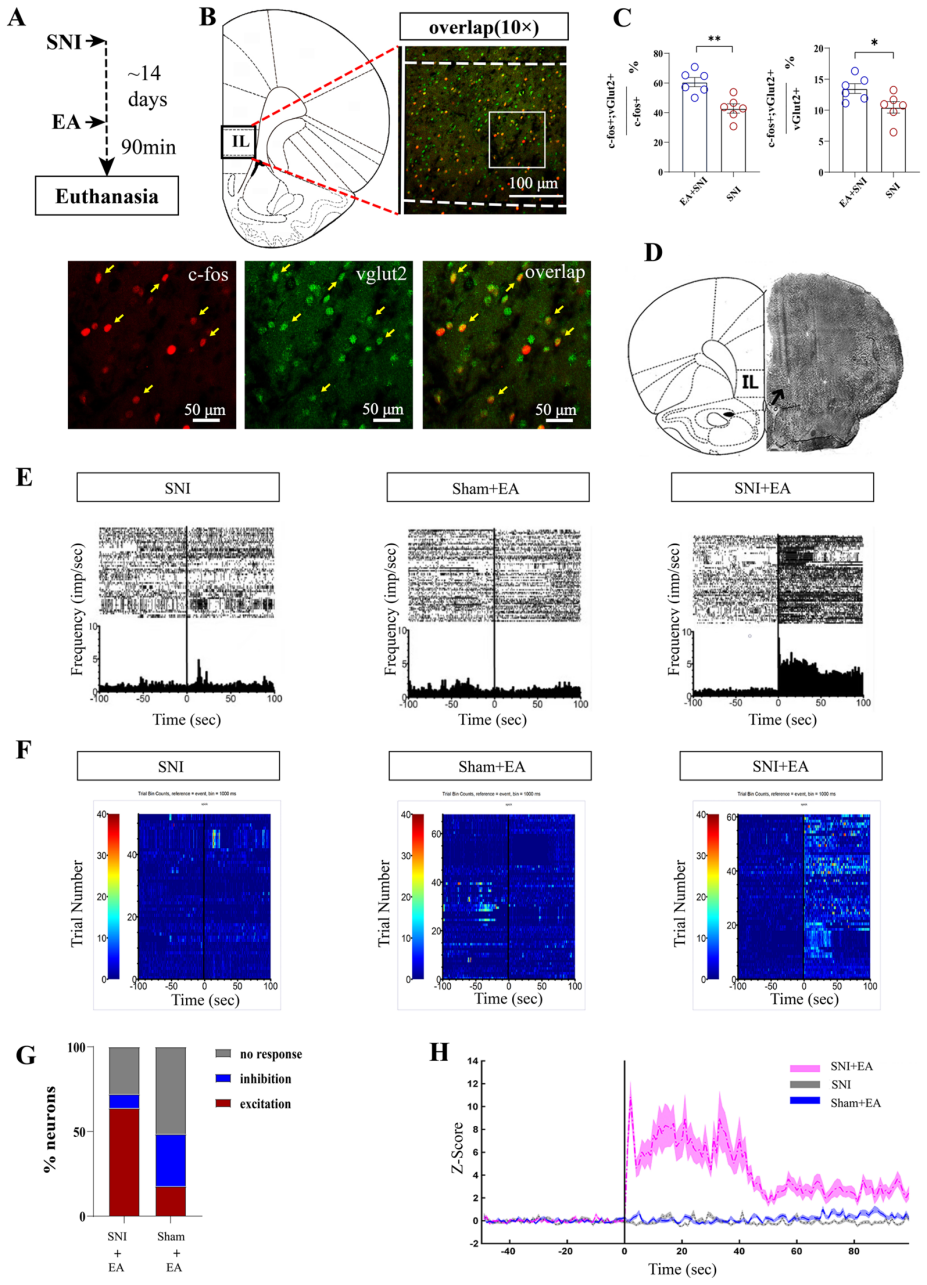
EA activates glutamatergic neurons in the IL in rats with pain. **A** Experimental strategy for immunofluorescence staining. **B** Representative photomicrograph showing that EA increased the number of c-Fos+/vglut2+ neurons colocalized in the IL. Top: scale bar, 100 μm. Bottom: magnified image showing the morphology of labeled neurons in the boxed area. **C** Quantification of vglu2+/c-Fos+ neuron colocalization in the IL. n = 6 rats/group. Two-tailed unpaired t test. The data are expressed as the means ± S.E.M.s. *P < 0.05; **P < 0.01. The experiment was repeated three times with similar results. **D** Photograph of a coronal section showing the IL recording electrode track. **E** Single-neuron examples of the prevalent response to EA before (− 100 to 0 s) and after EA stimulation (0–100 s) in the IL. **F** Z scored activity for all recorded IL putative glutamatergic pyramidal cells. n = 52 cells from 6 SNI group rats; n = 68 cells from 6 sham-operated (sham) group rats; n = 61 cells from 6 SNI + EA group rats. **G** Proportions of putative glutamatergic pyramidal neurons in the IL exhibited significant excitation or inhibition in response to EA stimulation in SNI rats or sham-operated (sham) rats. Among the 61 neurons, 39 (63.9%) exhibited excitation, 5 (8.2%) exhibited inhibition, and 17 (27.9%) exhibited no response in SNI rats. Among the 68 neurons, 12 (17.7%) exhibited excitation, 21 (30.8%) exhibited inhibition, and 35 (51.5%) exhibited no response in sham-operated rats. Chi-square tests were used. χ^2^ = 4.96, *P* = 0.01. **H** Mean Z score activity for IL putative glutamatergic pyramidal neurons before (− 40 to 0 s) and after EA stimulation (0–100 s) in SNI rats or sham-operated (sham) rats, displayed as the mean ± S.E.M. Two-way repeated-measures ANOVA, F = 16.5, *P* < 0.001

Pyramidal neurons in the mPFC produce glutamate as a neurotransmitter, with glutamatergic neurons being the major projecting neurons in the IL. We hypothesized that glutamatergic projecting neurons expressing vesicular glutamate transporter 2 (vGlut2) in the IL could play a central role in EA-mediated modulatory effects on pain processing. To confirm this, we performed c-Fos/vGlut2 double labeling to examine whether glutamatergic projections were activated in SNI rats following EA (Fig. 3A). EA stimulation on day 14 after SNI modeling increased number of colocalized c-Fos-immunopositive (c-Fos +) and vGlut2-immunopositive (vGlut2+) neurons in the IL (Fig. 3B, C). These data demonstrate that EA stimulation increases c-Fos expression preferentially in IL^Glu^ neurons in rats with pain.

Next, we recorded the activity of IL neurons using multichannel unit-recording before and after EA in SNI rats (Fig. 3D–H). IL neurons are categorized into putative glutamatergic pyramidal cells and interneurons on the basis of their firing rates and spike widths [28]. Consistent with previous reports [36], one cluster contained the majority of neurons with low firing rates (< 15 Hz) and broad spike waveforms (> 225 ms; putative excitatory pyramidal neurons), whereas the other contained neurons with high firing rates (> 15 Hz) and narrow spike waveforms (< 225 ms; putative inhibitory interneurons, Fig. S7). A total of 202 well-isolated single units were obtained from 15 rats. Classification was based on spike width and firing rate: units with spike width > 225 μs and firing rate < 15 Hz were classified as putative pyramidal (glutamatergic) neurons (n = 161, 79.7%). Units with spike width < 225 μs and firing rate > 15 Hz were classified as putative interneurons (n = 41, 20.3%). We quantified the impact of EA on both population activity (Z-score) and individual neurons in SNI or sham operated rats. In SNI rats, 63.9% of IL putative pyramidal neurons were excited by EA stimulation, whereas in sham-operated rats, 17.7% of IL neurons were excited by EA stimulation (Fig. 3E–G). Overall signaling strength, measured by the mean z-score, was significantly greater in the EA stimulated groups than at baseline (z = 5.28; *P* < 0.001; Fig. 3H). The increase in the activity of IL neurons induced by EA lasted at least 100 s. Conversely, in sham operated rats, there were no significant differences in signaling strength before or after EA stimulation (z = 1.49; *P* > 0.05; Fig. 3H). These results indicate that IL neural responses to EA stimulation are dependent on the animal’s state of pain, which is consistent with our behavioral and immunofluorescence results.

### Optogenetic stimulation of glutamatergic neurons in the IL mimics the effects of EA in SNI mice

**Fig. 4:**
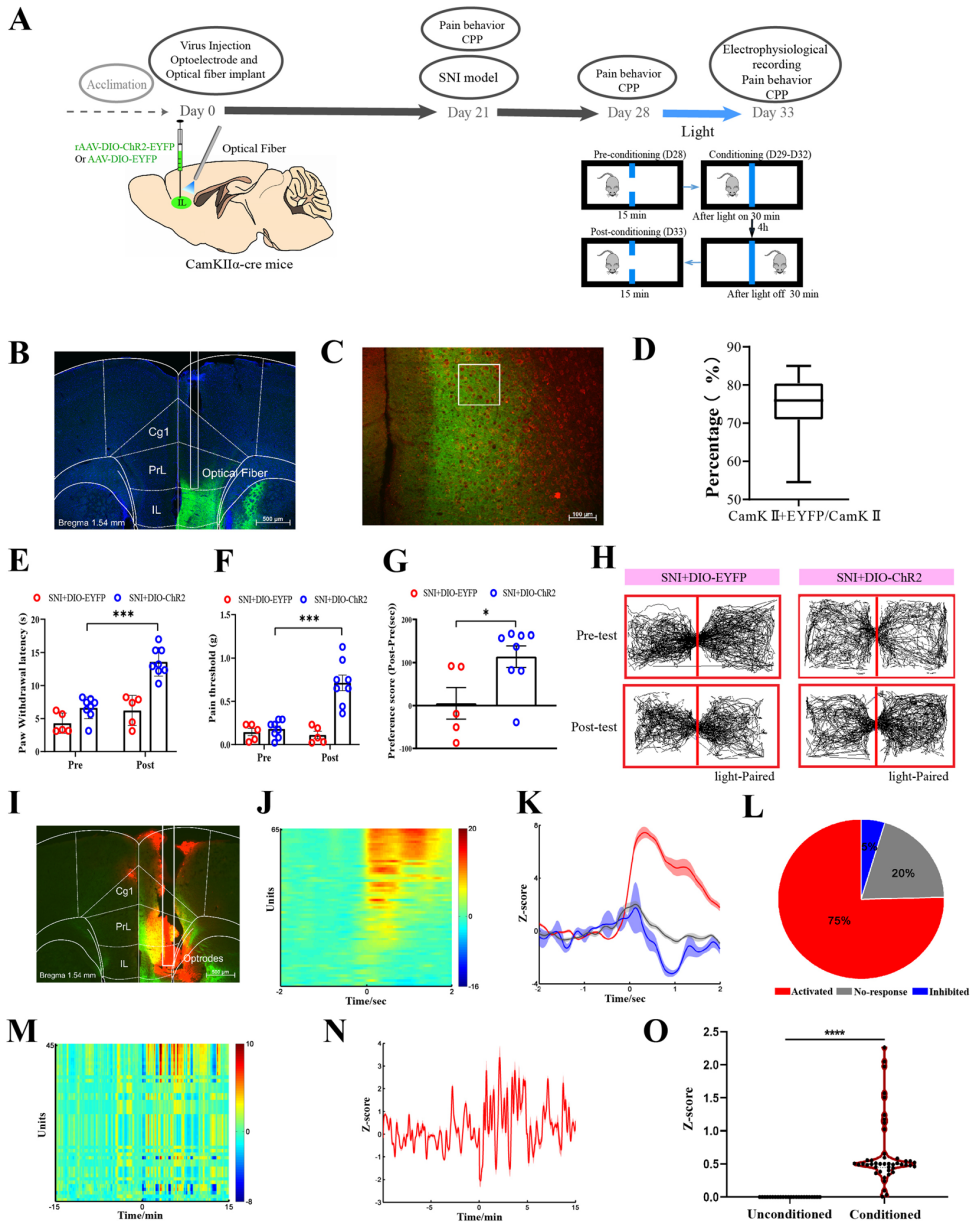
Optogenetic activation of glutamatergic neurons in the IL mimics the effect of EA in SNI animals. **A** Schematic diagram showing the timeline of the experiment. **B** Representative images showing the virus injection site and fiber embedding site in CamKIIα-Cre mice. Bar = 500 μm. **C** Colocalization of ChR2-EYFP+/CamKII+ neurons in the IL of mice. Bar = 100 μm. White squares indicate a fixed area of the IL of each section, and the analysis software counted stained nuclei within the area. Green: ChR2-EYFP expression; Red: CamKIIα immunostaining; Yellow: merged signal indicating colocalization. **D** Quantification of the percentage of ChR2-EYFP+/CamkII+ neurons colocalized with all CamkII+ neurons in the IL. The data are presented as the median ± interquartile range (IQR). **E** Paw withdrawal latency (s) before and after light stimulation (F (1, 11) = 12.16, *P* = 0.005). **F** Pain threshold (g) before and after light stimulation (F (1, 11) = 23.76, *P* < 0.0001). **E–F** Two-way ANOVA with Sidak's multiple comparisons test was used. n = 5–8 mice/group. **G** The CPP score. Two-tailed unpaired t test. t = 2.533, *P* = 0.028. **H** Trajectory of SNI mice in the CPP apparatus. Data (**E–G**) are presented as the mean ± S.E.M. **I** Schematic representation showing the viral injection site and optimal embedding site. Bar = 500 μm. **J** Heatmap of the Z score; each row represents one neuron. **K** Average Z scores for neurons with significantly increased (red, *P* < 0.0001) and decreased (blue, *P* < 0.0001) activity and no significant change (gray, *P* > 0.05). **L** The proportions of increased, decreased and not significantly changed neurons. Chi-square tests. χ^2^ = 7.96, *P* < 0.01. **M** Heatmap of the Z scores of all recorded units sorted by the mean Z score FR in paired or unpaired compartments; each row represents one neuron. **N–O** The average Z scores of the FRs of neurons in the IL. Data are presented as the median ± interquartile range (IQR). Two-tailed unpaired t test. t = 3.495. *P < 0.05, **P < 0.01, ***P < 0.001. ****P < 0.0001

Given that the above data are purely associative, they do not reflect whether increased neuronal activity is a cause or a consequence of EA effects. To further determine the causal role of the activation of IL^Glu^ neurons in pain and affective-motivational behaviors, a Cre-dependent rAAV expressing channelrhodopsin-2 (ChR2) fused with enhanced yellow fluorescent protein (rAAV-DIO-ChR2-EYFP) was injected into CamKIIα-Cre mice, and an optical fiber was implanted above the injection site to manipulate glutamatergic neurons (Fig. 4A, B). CamKIIα is widely expressed in excitatory pyramidal neurons in the cortex, including the IL, and is commonly used for genetic access to glutamatergic populations [62]. We found that approximately 80% of ChR2-EYFP-positive (ChR2-EYFP+) neurons overlapped with CamKII-immunopositive (CamKII+) neurons (Fig. 4B–D). The mice were stimulated with 473 nm blue light paired with one chamber of the CPP apparatus. Stimulation of the IL with blue light increased the thresholds of mechanical allodynia and thermal hyperalgesia in SNI mice (Fig. 4E, F), suggesting that the excitation of IL^Glu^ neurons could produce analgesic effects, which is consistent with previous studies [15, 17]. Interestingly, delivery of blue light in the IL resulted in a robust preference for the chamber paired with light stimulation in CamKIIα-Cre mice with SNI (Fig. 4G, H). These results indicate that the analgesic and CPP effects promoted by the optogenetic activation of IL^Glu^ neurons were similar to those of EA stimulation.

Next, we used in vivo electrophysiology combined with optogenetics to further assess whether the firing rate of IL neurons increased with time in the blue light-conditioned chamber in SNI mice. The same Cre-dependent rAAV approach was used and an optrode was implanted at the injection site to manipulate glutamatergic neurons (Fig. 4I). We observed that the mean Z-score was significantly greater following light stimulation (Fig. 4J–O), and 75% of the recorded IL neurons were activated during the photostimulation period (Fig. 4L), confirming the effectiveness of our approach. We then used the CPP paradigm to observe how the firing rates of IL neurons responding to light on the light-paired side or the nonpaired side changed after SNI mice received continuous conditioning with CPP for 4 days. The results revealed that the firing rate of IL neurons paired with the light-matched side was significantly increased, as indicated by population activity (Z-score) and individual neuronal activity in SNI mice. These results suggest that the activation of IL^Glu^ neurons is involved in light-induced analgesia and CPP behavior in SNI mice.

Inhibition of glutamatergic neurons in the IL reverses the effects of EA in SNI mice. We reasoned that if the activation of IL^Glu^ neurons mimics the effects of EA-induced peripheral neuromodulation, then the inhibition of these cells should reverse EA-induced CPP. To test this, we first determined the causal role of IL inactivation in EA-induced CPP via IL microinjection of a mixture of muscimol + baclofen (M/B, GABAA + GABAB agonists) to nonselectively inactivate most IL neurons 5–10 min prior to EA treatment in SNI rats (Figure S8A). We found that the increases in CPP scores elicited by EA stimulation were ablated by transient IL inactivation (Figure S8). These results implicate the role of IL neurons in EA-induced CPP behavior in SNI rats.

**Fig. 5:**
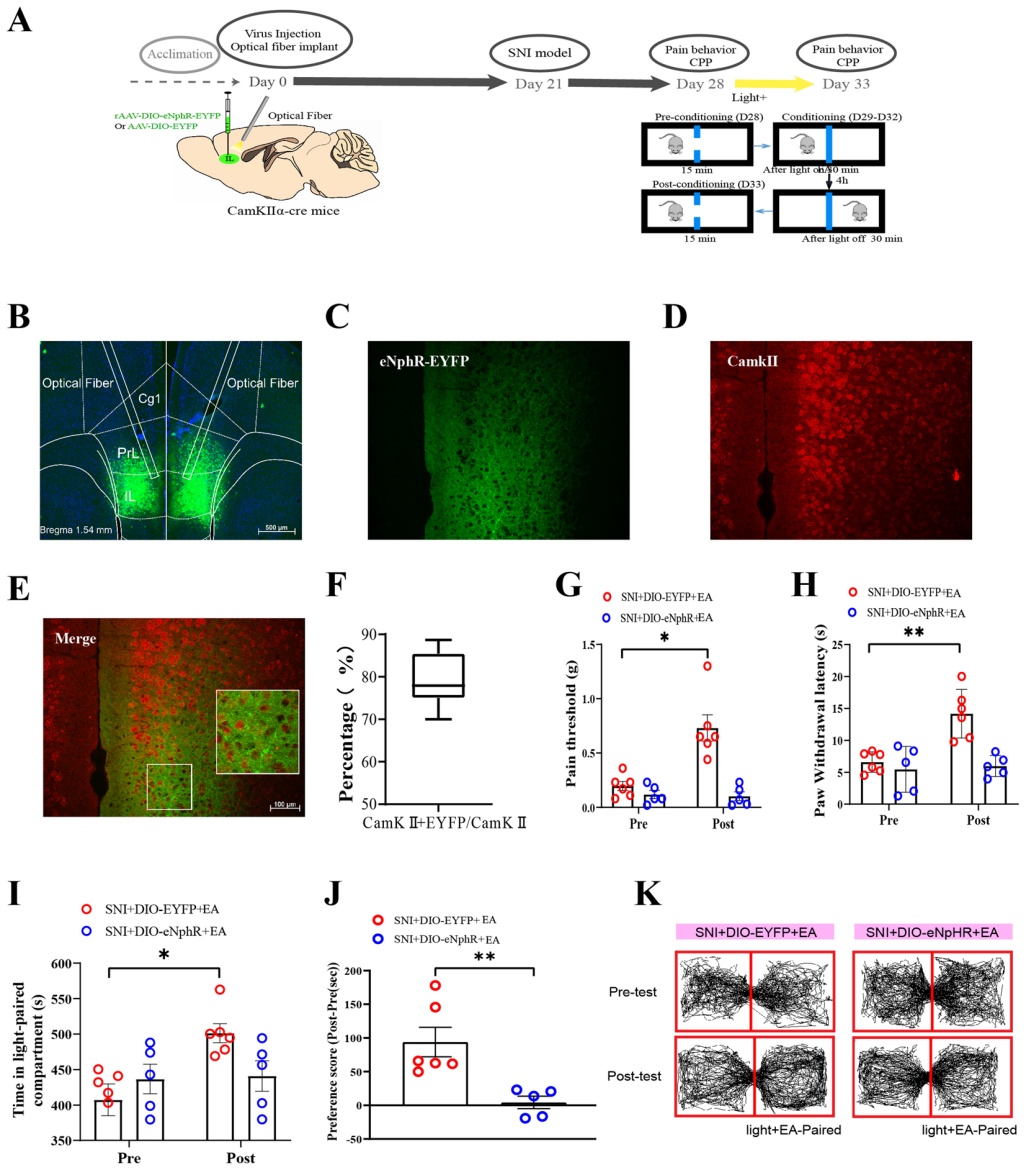
Inhibition of glutamatergic neurons in the IL reverses the effects of EA in SNI mice. **A** Schematic diagram showing the timeline of the experiment and the injection of rAAV-EYFP or rAAV-eNpHR-EYFP into the bilateral IL and the embedding of optical fibers in CamKIIα-Cre mice. **B** Representative images of the virus injection site and fiber embedding site in CamKIIα-Cre mice. Bar = 500 µm. **C** Representative photomicrograph showing the expression of eNpHR-EYFP in the IL. **D** Representative photomicrograph showing CamKII-stained neurons. **E** Colocalization of eNpHR-EYFP+/CamkII+ neurons in the IL of mice. Bar = 100 µm. **F** Quantification of the percentage of eNpHR-EYFP+/CamkII+ neurons colocalized with all CamkII+ neurons in the IL. The data are presented as the median ± interquartile range (IQR). **G–I** The increased thresholds of mechanical allodynia and thermal hyperalgesia induced by EA were inhibited after yellow light stimulation. Two-way ANOVA with Sidak's multiple comparisons test was used. n = 5–6 mice/group. **G** Pain threshold (F (1, 9) = 10.34, *P* = 0.01). **H** Paw withdrawal latency (s) (F (1, 9) = 12.23, *P* = 0.007). **I** Time in the light-paired compartment (s) before and after light stimulation (F (1, 9) = 12.21, *P* = 0.007). **J** CPP scores. Two-tailed unpaired t test. t = 3.495, *P* = 0.007. **K** Trajectory of SNI mice in a CPP apparatus. All the data are presented as the means ± S.E.M.s. *P < 0.05, **P < 0.01

We further tested whether optogenetic inhibition of glutamatergic neurons in the IL reversed the effects of EA in SNI mice. A Cre-dependent rAAV expressing eNpHR fused with enhanced yellow fluorescent protein (rAAV-DIO-eNpHR-EYFP) was injected into CamKIIα-Cre mice, and an optical fiber was implanted at the injection site to inhibit glutamatergic neurons via 589 nm yellow light stimulation. We found that approximately 80% of eNpHR-EYPF+ neurons overlapped with CamkII+ neurons (Fig. 5F). Yellow light (5–8 mW, constant) was delivered into the IL while SNI mice were receiving EA stimulation (Fig. 5B–F). We observed that the positive effects of EA on thresholds of mechanical allodynia and thermal hyperalgesia were inhibited during simultaneous yellow light stimulation (Fig. 5G, H), indicating that the inhibition of IL^Glu^ neurons reversed the analgesic effects of EA. Furthermore, EA-induced CPP (increased CPP scores) was ablated by delivery of yellow light in the IL region (Fig. 5I–K). These results clearly demonstrate that glutamatergic neurons in the IL are necessary for EA-induced pain relief and CPP behaviors.

### IL to NAc shell pathway mediates EA-induced pain relief and affective-motivational behaviors

**Fig. 6:**
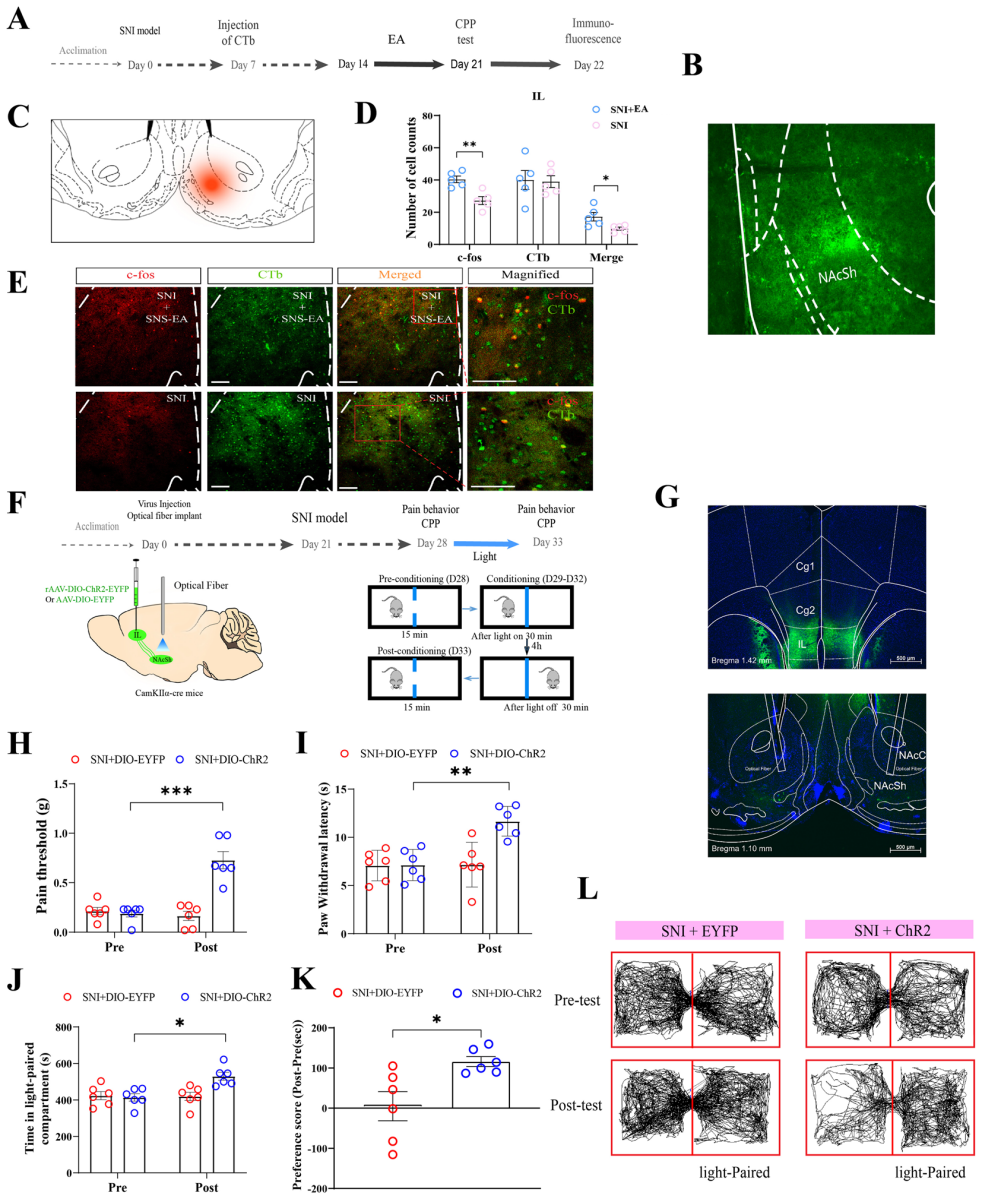
IL to NAc shell activation is necessary for EA-induced pain relief and CPP behaviors. **A** Schematic diagram showing the timeline of the experiment. **B** Fluorescent photomicrographs of representative injection sites in the NAc shell. **C** Schematic drawings through the NAc shell showing the location of CTb injections in SNI rats. **D** Numbers of c-Fos+, CTb+, and CTb+/c-Fos+ neurons colocalized in the IL. Two-tailed unpaired t test. n = 5 rats/group. For c-Fos+, t = 4.113, *P* = 0.003; for CTb+, t = 0.145, *P* = 0.889; and for CTb+/c-Fos+, t = 2.603, *P* = 0.032. **E** Representative photomicrograph showing CTb+/c-Fos+ neuron colocalization in the IL. Scale bar, 75 mm. Magnified scale bar, 100 mm. **F** Schematic diagram showing the timeline of the experiment and the injection of rAAV-EYFP or rAAV-ChR2-EYFP into the IL and the embedding of optical fibers into the NAc shell in CamKIIα-Cre mice. **G** Schematic representation showing the viral injection site in the IL (top) and the fiber embedding site in the NAc shell (bottom). Bar = 500 μm. **H–I** The thresholds of mechanical allodynia and thermal hyperalgesia were increased after blue light stimulation. Two-way ANOVA with Sidak's multiple comparisons test was used. n = 6 mice/group. **H** Pain threshold (F (1, 10) = 29.48, *P* = 0.0003). **I** Paw withdrawal latency (s) (F (1,10) = 8.19, *P* = 0.017). **J** Time in the light-paired compartment (s) before and after light stimulation (F (1, 10) = 11.99, *P* = 0.006). **K** CPP scores. Two-tailed unpaired t test. t = 2.916, *P* = 0.031. **L** Trajectory of SNI mice in the CPP apparatus during the pretest phase and posttest phase. The data are presented as the means ± S.E.M.s. *P < 0.05, **P < 0.01, ***P < 0.001

The contributions of IL outputs to pain relief and affective‒motivational responses induced by EA remain undiscovered. The NAc is a good candidate that is known to receive glutamatergic inputs from the mPFC, process pain signals and integrate the rewarding valence in the descending pathway [37, 38]. Previous anatomical studies have indicated that PL fibers are distributed massively throughout the core and shell regions, but in contrast, IL fibers project fairly selectively to the NAc shell [39, 40]. We determined whether the IL-NAc shell pathway could be activated by EA stimulation in SNI rats using CTb/c-Fos double-label immunofluorescence staining and injecting the retrograde tracer cholera toxin B subunit (CTB)-Alexa 488 into the NAc shell (Fig. 6A–E). Following a 1 week period, we detected retrogradely labeled neurons in the IL. Nearly 41.1% of the CTB-labeled NAc shell projecting IL neurons (green) expressed the Fos protein (red) in the EA-stimulated rats, whereas only 28.6% of the SNI group expressed the Fos protein (Fig. 6D). There was a significant difference between the EA and SNI groups in the percentage of CTB retrograde-labeled neurons that expressed Fos (*P* < 0.001, Fig. 6D, E), indicating a stronger connection in the IL‒NAc shell neuronal pathway following EA stimulation.

To further investigate whether the IL‒NAc shell pathway is required for EA-induced pain relief and affective and motivational responses, we injected ChR2- or EYFP-expressing rAAVs into the IL and placed optical fibers immediately above the NAc shell in mice (Fig. 6F, G). Like the effects of activating the IL, optogenetic activation of terminal IL projections to the NAc shell strongly induced analgesic effects on mechanical allodynia and thermal hyperalgesia, leading to a decrease in mechanical and thermal hypersensitivity (Fig. 6H, I). In contrast, light stimulation of the NAc shell did not have any observable analgesic effects on the mice injected with EYFP-expressing rAAVs (Fig. 6H, I). Furthermore, blue light was delivered to the NAc shell during the conditioning phase of CPP. After 4 days of conditioning, SNI mice injected with ChR2-expressing rAAVs showed a robust preference for the chamber paired with light stimulation compared with the results with no light-paired chamber (Fig. 6J–L).

We next used chemogenetic approach to further confirm the effect of inactivating the IL^Glu^-NAc shell pathway on EA-induced analgesia and affective-motivational behaviors. We bilaterally injected the IL of C57BL/6J mice with a rAAV-EF1a-DIO-hM4D(Gi)-EGFP-WPREs (DREADDs, hM4Di) or rAAV-EF1a-DIO- EGFP-WPRE-hGH polyA as a control. A rAAV-CaMKlla-CRE-WPRE-hGH PA was bilaterally injected into the NAc shell (Figure S9A, B). As expected, the increased paw withdrawal latency and pain threshold induced by EA were attenuated by intraperitoneal injection of the hM4Di agonist clozapine-N-oxide (CNO) (Figure S9C). CNO injection also significantly reduced the EA-induced preference of SNI model mice (Figure S9D). In addition, we also evaluated the impact of inactivating IL^Glu^-NAc shell pathway on EA-induced anxiolytic effects in SNI mice using the OFT and the EPM (Figure S9E–I). Anxiolytic-like effects induced by EA stimulation were blunt by the CNO, as indicated by the decreased distance traveled in the center area and open arms during OFT and EPM tests, respectively. These results demonstrate that IL^Glu^ to NAc shell pathway mediates EA-induced pain relief and affective-motivational behaviors.

### GABAergic neurons in the NAc shell are involved in EA-induced analgesia and affective-motivational behaviors

**Fig. 7:**
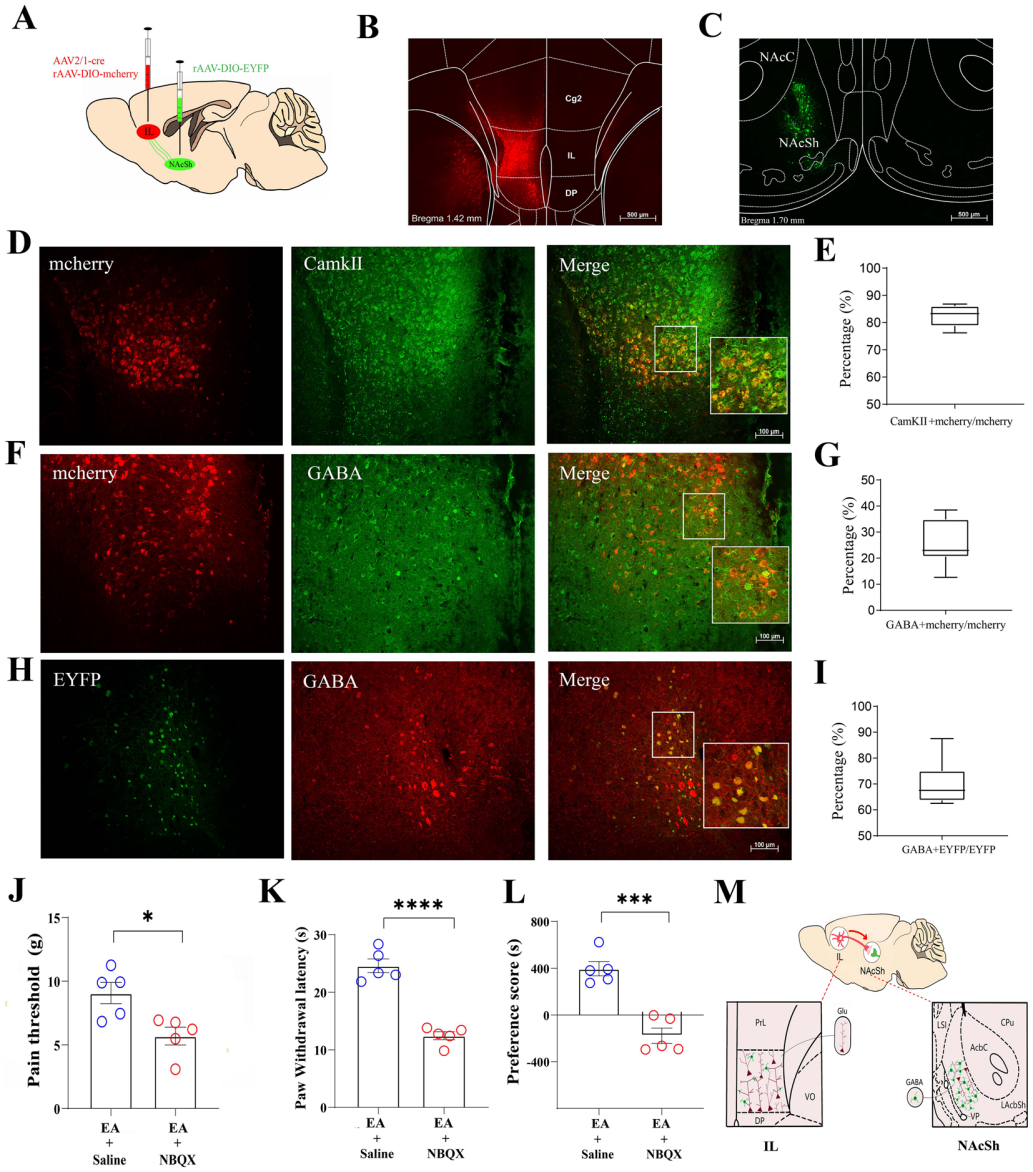
IL^Glu^ to the NAc shell^GABA^ circuit is involved in EA-induced pain relief and affective-motivational behaviors. **A** Schematic showing the injection of AAV2/1-cre and rAAV-DIO-mCherry into the left IL and rAAV-DIO-EYFP into the left NAc shell in C57BL/6J mice. **B, C** Typical image of viral infusion in the IL (**B**) and NAc shell (**C**). Bar = 500 μm. **D** Representative images of mCherry expression, CamkII immunostaining, and colocalization in the IL. Bar = 100 μm. **E** Quantification of the percentage of mCherry+/CamkII+ neurons colocalized with all mCherry+ neurons in the IL. **F** Representative images of mCherry expression, GABA immunostaining and colocalization in the IL. Bar = 100 μm. **G** Quantification of the percentage of mCherry+/GABA+ neurons colocalized with all mCherry+ neurons in the IL. **H** Representative images of EYFP expression, GABA immunostaining and colocalization in the NAc shell. Bar = 100 μm. **I** Quantification of the percentage of EYFP+/GABA+ neurons colocalized with all EYFP+ neurons in the NAc shell. Data (**E, G, I**) are presented as the median ± interquartile range (IQR). **J, L** Intra-NAc shell infusion of NBQX blocked the analgesic effects and CPP of EA in SNI rats. Two-tailed unpaired t test. n = 5 rats/group. **J** Pain threshold (t = 3.108, *P* = 0.0145). **K** Paw withdrawal latency (t = 8.735, *P* < 0.0001). **L** CPP scores (t = 3.766, *P* = 0.006). *P < 0.05, **P < 0.01, ****P < 0.001. All the data are presented as the means ± S.E.M.s. **M** Scheme depicting the ILGlu-to-NAc shellGABA pathway involved in EA-induced analgesia and CPP

To elucidate the precise cell type-specific organization and connection between the IL and NAc shell, we first identified which type of neurons in the NAc shell the IL projected to. C57BL/6J mice were injected with a monosynaptic transneuronal virus (AAV2/1-Cre) and a Cre-dependent mCherry fluorescent protein virus (AAV-DIO-mCherry) into the left IL and a Cre-dependent EYFP fluorescent protein virus (AAV-DIO-EYFP) into the left NAc shell (Fig. 7A–C). With respect to the expression of mCherry, we observed a localized transfection of the AAV2/1-Cre virus in the IL brain region. Furthermore, we found many of overlapping mCherry+ neurons with CamKII+ neurons (Fig. 7D, E) in the IL and only a few with GABA+ neurons (Fig. 7F, G). In addition, we observed that EYFP+ neurons from the IL projections to the NAc shell had approximately 70% co-expression with GABA+ neurons (Fig. 7H, I), confirming the presence of an IL^Glu^ to NAc shell^GABA^ projection (Fig. 7M).

α-Amino-3-hydroxy-5-methyl-4-isoxazole propionic acid (AMPA) receptors are the primary glutamate receptors in the NAc and are crucial for pain and affective behaviors [27, 42]. To confirm the role of NAc AMPA receptors as specific targets of the NAc shell for EA stimulation, we infused NBQX, a highly specific antagonist of AMPA receptors, into the NAc shell during EA stimulation. We found that NBQX blocked the antinociceptive effects of EA, including mechanical and thermal hyperalgesic changes (Fig. 7J, K). These results suggest that AMPA receptors in the NAc shell can be targeted or recruited during EA stimulation to relieve pain. Furthermore, we assessed whether these receptors are involved in EA-induced CPP in SNI rats. Indeed, intra-NAc shell infusion of NBQX significantly ablated EA-induced CPP (Fig. 7L). These results provide evidence that AMPA receptors in the NAc shell are important molecular targets of EA.

Subunit trafficking plays an important role in providing long-term plasticity of excitatory neurotransmission through AMPA receptors [41]. Robust evidence indicates that persistent pain alters AMPA receptor subunit levels in the NAc [42, 43]. Herein, we investigated whether EA could increase AMPA receptor trafficking in the NAc shell. We isolated synaptoneurosome fractions from the NAc shell and measured GluR1 and GluR2 subunit levels in SNI rats after 3 days of EA stimulation. The levels of these subunits remained unchanged after EA stimulation (Figure S10). These results indicate that EA does not necessarily alter the synaptic levels of AMPA receptors in a meaningful manner. We posit that the effects of EA are likely attributed to the biophysical functions of AMPA receptors, rather than to long-term changes in receptor composition.

Next, to determine whether GABA signaling in the NAc shell is involved in the effects of EA, we explored the protein expression of GABA in SNI rats following EA using c-Fos/GABA double labeling. EA stimulation increased c-Fos activation in GABAergic cells belonging to the NAc shell (Fos+ and GABA+ neurons in the NAc shell in EA rats, 71 ± 9%; and in SNI rats, 54 ± 5%) on day 14 after SNI modeling (Figure S11). The percentage of c-Fos+/GABA+ neurons colocalized in the NAc shell in SNI rats was not significantly different from that in control rats (Figure S11A). In addition, EA did not affect the percentage of c-Fos+/GABA+ neurons colocalized in the NAc core (55 ± 7% for SNI vs 51 ± 3% for SNI + EA). These results showed that EA stimulation specifically activated GABAergic neurons in the NAc shell, but not in the NAc core.

### EA induces affective-motivational responses after pain relief in patients with chronic low back pain (cLBP)

**Fig. 8:**
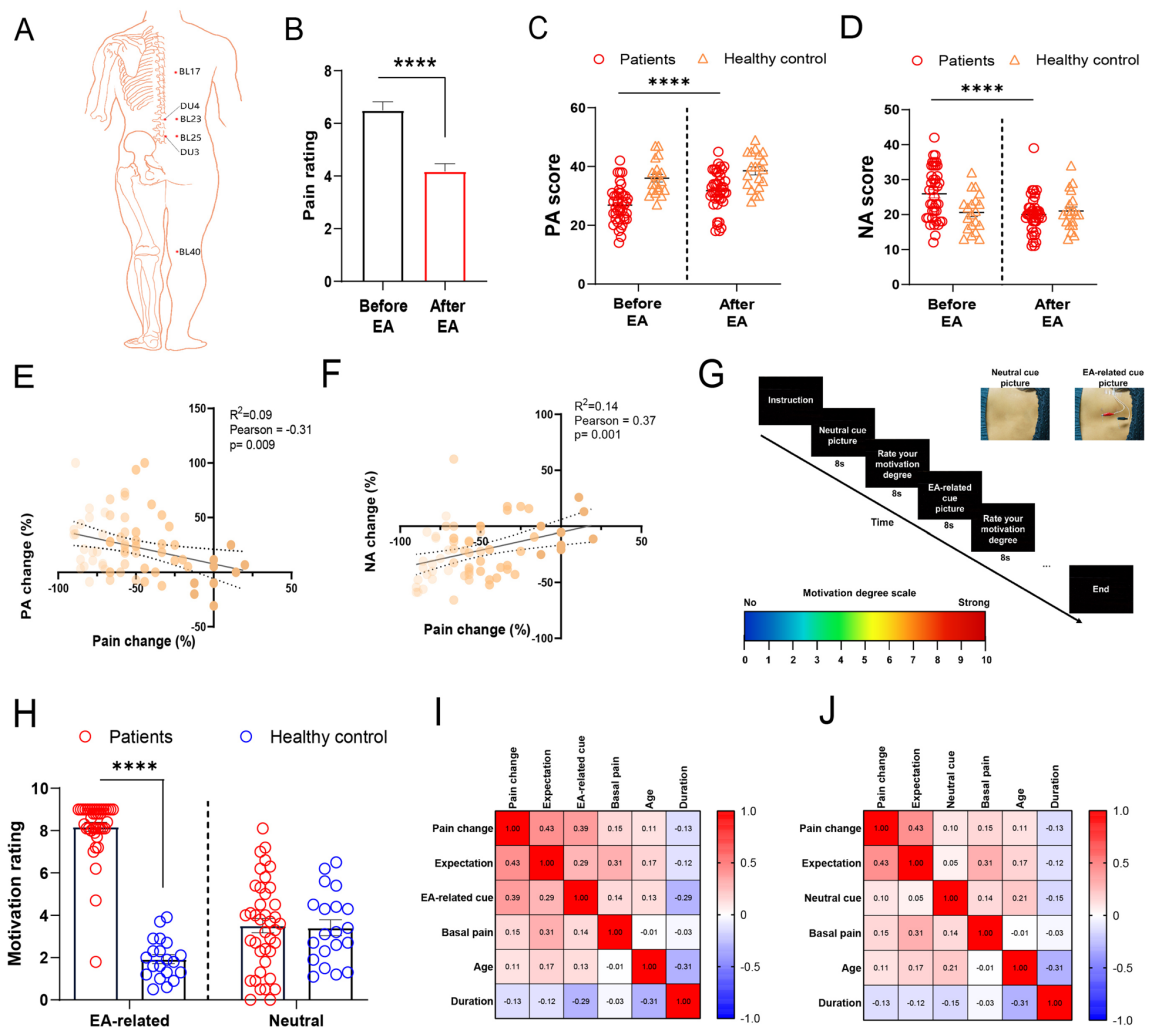
EA induces affective‒motivational responses after pain relief in patients with chronic low back pain (cLBP). **A** Acupuncture points used to treat cLBP. **B** VAS score of pain before and after EA treatment in cLBP patients. Two-tailed unpaired t test. n = 40 patients. t = 5.322, *P* < 0.0001. **C** Positive affect (PA) scores before and after EA treatment (F (1, 58) = 25.95, *P* < 0.0001). **D** Negative affect (NA) scores before and after EA treatment (F (1, 58) = 15.894, *P* < 0.0001). **E** The correlation between changes in the VAS pain rating and changes in the PA score after EA treatment. r = − 0.3142, *P* = 0.009. **F** The correlation between changes in the VAS pain rating and changes in the NA score after EA treatment (r = 0.3753, *P* = 0.002). A series of Pearson product‒moment correlations were performed (**E, F**). **G** Experimental sequence. All participants (40 patients and 20 healthy volunteers) viewed a total of 20 images (10 EA-related and 10 neutral) in a fixed semirandom order, blocked, and reported how much they wanted to receive EA treatment after each image on an 11-degree numerical rating scale after EA. Two sample images presented to subjects depicting neutral (upper left) and EA-related (upper right) levels. **H** Motivational responses induced by EA-related cues after EA treatment (F (1, 58) = 90.97, *P* < 0.0001). **I, J** A total of patients with cLBP were used to calculate all bivariate Pearson correlations (two-tailed) when exposed to EA-related cues (**I**) or neutral cues (**J**). The data are shown as the means ± S.E.M.s. Two-way ANOVA with Sidak's multiple comparisons test (**C, D, H**). n = 40 patients; n = 20 healthy controls. *****P < 0.0001

To examine whether EA induced affective‒motivational responses following pain relief in humans, 40 patients with cLBP (26 females; mean age 41.7 years) were included in the analysis of affect and motivation after EA treatment. Additionally, 20 healthy volunteers with matched demographic characteristics were recruited to constitute the control group (Table S1). Separate 10-point visual analog scales (VASs) were used for assessment of pain before and after EA treatment. The Positive and Negative Affect Scale (PANAS) was used to assess affective responses (see Supplementary Methods for detail). The acupuncture points selected in the present study were based on systematic reviews, consensus meetings with clinical experts, and our clinical experience (Fig. 8A). EA significantly reduced the pain intensity rating, with a 52% decrease (Fig. 8B). EA induced higher positive affect (PA) scores and lower negative affect (NA) scores in patients with cLBP (Fig. 8C, D). Changes in the VAS pain rating were significantly correlated with changes in the PA scores and NA scores after EA stimulation in patients with cLBP (Fig. 8E, F). In healthy volunteers, EA did not affect PA or NA scores (Fig. 8C, D), indicating that EA may not elicit an affective response in the absence of pain, which is consistent with preclinical data.

To assess motivation to respond to EA-related cues in the laboratory, participants were asked to state their motivation for two categories of slides (EA and neutral) on the VAS (Fig. 8G, see Supplementary Methods for details) after EA stimulation. Two-way mixed-factorial ANOVA revealed significant main effects of group. Patients with cLBP showed increased ratings of motivation for EA-related slides compared with healthy volunteers after EA stimulation (Fig. 8H). There were no significant differences between patients and healthy controls in terms of neutral pictures. When patients were exposed to EA-related cues, significant correlations were obtained between ratings of motivation and pain relief or the expectation of pain relief in patients with cLBP (Fig. 8I). When participants were exposed to neutral-related cues, no significant correlations were detected between the ratings of motivation and pain relief (Fig. 8J).

## Discussion

Peripheral neuromodulation, particularly EA, can be considered a highly specialized sensory experience. It can be precisely localized in terms of somatotopy and described in different modalities and intensities [46]. Local sensory signals induced by EA recruit a basic sensory mechanism that leads to the stimulation of peripheral nerve terminals, which carry processed information to many brain regions. In this study, we systematically examined the affective‒motivational responses of pain relief following the application of EA in human and animal models in the context of pain. We illustrate critical behavioral changes evoked by EA treatment, therefore impacting our understanding of EA-induced affective and motivational responses. EA not only produces an analgesic effect but also evokes profound affective and motivational changes (CPP, anxiolytic and antidepressive effects) after pain relief. Our data emphasize that EA neuromodulation of affective and motivational processes may be dependent on the animal’s state of pain. EA may increase motivation in the presence of pain but is not related to intrinsic motivational aspects. These results provide a proof-of-concept basis that corroborates the idea that peripheral neuromodulation signals reflect a multidimensional composite created by afferent sensory inputs and that their processing in the brain circuitry modulates expectations and affective and motivational responses. Hence, EA not only is a sensory input but also represents a dynamic state that shapes diverse psychosocial responses. These results may provide insights into the contribution of EA to multiple clinical applications, including the treatment of pain conditions with psychiatric comorbidities, as well as improve our understanding of EA-related placebo effects.

Acupuncture or EA has been shown to improve negative emotions in various diseases, including pain-related disorders [12, 15, 47]. In the present study, we illustrate critical positive emotions and enhanced motivation evoked by EA, therefore impacting our understanding of EA-induced affective and motivational responses. EA-induced motivational responses of pain relief are clinically significant [48]. cLBP is a prevalent chronic pain condition with well-documented affective and motivational components, and often exhibit neuropathic-like features [63, 64]. Kong et al. previously suggested that conditioning positive expectations can amplify acupuncture analgesia, an observation that was detected by subjective pain sensory rating changes [49]. Our results revealed that EA analgesia induced CPP in different animal models of pain, indicating that affective and motivational responses may evoke patients’ inherent power to alter the degree and quality of pain that is experienced and drive decisions to seek future treatments. These data are consistent with our clinical observation that a patient sought EA even after the disappearance of pain symptomatology. In addition, our data emphasize that EA-induced peripheral neuromodulation of affective and motivational processes may be dependent on the animal’s state of pain. CPP has been induced by EA in different animal models of pain but not in sham-operated normal rats, suggesting that EA may increase motivation in the presence of pain but is not related to intrinsic motivational aspects. This conclusion is consistent with the concept of negative reinforcement elicited by the relief of an aversive state [48, 50]. Additionally, the results of our behavioral experiments support the conclusions of previous studies on the positive regulation of EA [33, 51]. The variability in responses to EA might be partially explained by the state-dependent effects of EA [52]. According to the theory of traditional Chinese medicine, the therapeutic actions of acupuncture are achieved by normalizing metabolism or pathogenic changes toward homeostasis. For example, peripheral neuromodulation with EA can decrease heart rate in tachycardic conditions while increasing heart rate in the context of sinus bradycardia. Interestingly, we observed that EA activated neural activity in the IL region of the mPFC in the context of acute pain but did not stimulate the activity of IL neurons in sham-operated animals, indicating that its cortical modulation is also pain state dependent. Cortical top–down output comes from the interaction between internally generated activity and sensory-driven factors [53]. Notably, EA did not alter the synaptic levels of AMPA receptors in the NAc shell in SNI rats, indicating that the effects of EA are likely attributed to the biophysical functions of neural circuits rather than to long-term changes in receptor composition. The present study highlights a cortical mechanism by which EA stimulation modulates pain in a state-dependent manner.

Neuromodulation alters central nervous system activity through the targeted delivery of stimuli to specific neurological sites. Indeed, brain imaging studies have demonstrated that EA can induce widespread changes in brain activity. In the present study, we identified a top–down neural circuit from the IL to the NAc shell that underlies EA-mediated analgesia and affective‒motivational responses. The IL plays pivotal roles in modulating goal-directed behaviors, emotional processes, and executive functions [54, 55]. IL activation reflects a form of externally elicited top–down control that modulates sensory and affective processes of pain [22, 56, 57]. Human and animal studies have shown that chronic pain induces decreased activity in the mPFC [16, 17, 57]. Stimulation of the mPFC produces descending pain control, including sensory and affective components [42]. Both our laboratory and others have demonstrated that acupuncture modulates neuronal activity within the IL [16, 23, 58]. Recent studies have also shown that the analgesia induced by EA is potentiated in association with neurostimulation in the IL [22]. In addition, we determined that the activation of IL glutamatergic neurons mimicked EA-induced analgesia and CPP behaviors, whereas their inhibition reversed the EA-mediated effects. Given that the clinical application of IL stimulation procedures such as deep brain stimulation (DBS) is limited by anesthetics and surgery, EA-induced peripheral neuromodulation may represent an alternative strategy for direct electrical IL stimulation as a means of activating the IL to treat symptoms associated with pain.

IL glutamatergic outputs project almost exclusively to the NAc shell [39, 40]. Activation of the IL‒NAc shell circuit has been shown to crucially regulate the sensory and aversive components of acute and chronic pain [20]. Our results indicated that glutamatergic inputs from the IL to the NAc shell were activated by EA stimulation. Moreover, the inhibition of glutamatergic neurons in the IL reversed EA-induced analgesia, CPP and anxiolytic-like behaviors, providing direct evidence that IL-NAc shell top–down control is a potential mechanism of EA-induced analgesia and affective-motivational processes. Previous studies have highlighted the contribution of the NAc shell to the mediation of pain, the affective-motivational value and the saliency of external stimuli [9, 28]. Notably, recent fMRI studies have demonstrated that significant functional responses in the NAc are associated with acupuncture analgesia [59]. Our study further expands these findings by indicating that neurons from the IL‒NAc shell pathway are coexpressed with approximately 70% of GABAergic neurons and that GABAergic neurons in the NAc shell are involved in EA-induced analgesia and affective and motivational processes. Some studies have demonstrated that GABA is responsible for the inhibition of pain signals in particular pathways [60], indicating the effects of GABA on descending pain modulation. A recent study revealed that GABAA receptors are involved specifically in the regulation of NAc neural activity [61]. Exogenous administration of GABA could attenuate the responses of “pain-excited neurons” modulated by GABAA receptors in the NAc and lead to an analgesic effect. In animal studies, ST36 is a well-standardized, commonly used acupoint for pain and affective studies, allowing for precise mechanistic investigation. In the clinical study, we selected acupoints commonly used for cLBP based on clinical guidelines and expert consensus. Given that previous fMRI studies have demonstrated that acupuncture increases activity in the mPFC [44] and that expectancy scores after EA treatment are significantly associated with increased NAc‒mPFC functional connectivity in patients with knee pain [45], our results provide evidence that EA can induce affective‒motivational responses and that this likely involves the activation of the mPFC‒NAc neural circuit. Moreover, although we included non-acupoint EA controls in several behavioral and immunofluorescence experiments (Figures S6), future studies would benefit from systematically incorporating non-acupoint stimulation across all experimental modalities—including optogenetic, chemogenetic, and circuit-specific manipulations—to more comprehensively dissect the acupoint-specific versus general neuromodulatory contributions of EA. This would further clarify whether the observed effects are unique to ST36 stimulation or may also be elicited by generalized peripheral electrical stimulation. In addition, the potential discrepancies in different acupoints should be taken into account in the subsequent studies.

## Conclusions

In conclusion, our data from humans and animals provide strong evidence that peripheral neuromodulation with EA induces analgesia and affective‒motivational responses. EA-induced neuronal activity in the IL^Glu^ to NAc shell^GABA^ pathway can drive EA-induced analgesia and affect-motivational processes. These results illustrate an example in which the emotional dimension of pain is directly influenced through the peripheral neuromodulation evoked by EA and provide the basis for the use of EA to precisely target top–down neural circuits to relieve chronic pain in psychological and clinical situations.

## Supplementary Information

Additional file 1.

## Data Availability

The key data needed to evaluate the conclusions in the paper are presented in the paper and/or the Supplementary Materials. The datasets used and/or analyzed during the current study are available from the corresponding author on reasonable request.

## Abbreviations

EA: Electroacupuncture
CPP: Conditioned place preference
OFT: Open field test
EPM: Elevated plus maze
SNI: Spared nerve injury
IL: Infralimbic cortex
NAc: Nucleus accumbens
BLA: Basolateral amygdala
CeA: Central nucleus
LH: Lateral hypothalamus
VTA: Ventral tegmental area
Cg1: Cingulate cortex
PL: Prelimbic cortex
Glu: Glutamatergic
vGlut2: Vesicular glutamate transporter 2
GABA: γ-aminobutyric acid
mPFC: Medial prefrontal cortex
PWL: Paw withdrawal latency
MIA: Monosodium iodoacetate
INP: Incisional injury pain
SPT: Sucrose preference test
FST: Forced swimming test
DS: Difference score
CTB: Cholera Toxin Subunit B
M/B: Muscimol + baclofen
AMPA: α-Amino-3-hydroxy-5-methyl-4-isoxazole propionic acid
cLBP: Chronic low back pain
VAS: Visual analog scales
PANAS: Positive and Negative Affect Scale
PA: Positive affect
NA: Negative affect
DBS: Deep brain stimulation
