# Supplementary material for: A top–down neural circuit for affective-motivational responses of pain relief induced by electroacupuncture
Source: Chin Med. 2026 Mar 1;21:73. doi: 10.1186/s13020-026-01349-5 (PMC12950241; doi:10.1186/s13020-026-01349-5)
Supplement: Supplementary file 1 — Additional file 1. [file 13020_2026_1349_MOESM1_ESM.pdf]

Supplementary Material

A top-down neural circuit for affective-motivational responses of pain relief induced by electroacupuncture

Hui Liu et al

Corresponding author: Sheng Liu, liusheng@shutcm.edu.cn

This PDF file includes:

Key Resources Table
Supplementary Methods
Figures S1 to S11
Tables S1 to S2

Other Supplementary Materials for this manuscript include the following:

Original blots for Western blot analysis

KEY RESOURCES TABLE

REAGENT or RESOURCE	SOURCE	IDENTIFIER
Antibodies		
Anti-c-Fos (mouse）	Abcam	ab208942
Anti-vGlut2	Thermo Fisher Scientific	42-7800
anti-CamK II	Abcam	ab52476
Anti-GABA (rabbit)	Sigma	A2052
Goat Anti-Mouse	Abcam	ab150115
Normal Goat Serum	Abcam	ab7481
VGLUT2 Polyclonal Antibody	Thermo Fisher Scientific	PA5-77432
Donkey Anti-Mouse IgG H&L (Alexa Fluor® 488)	Abcam	ab150109
Donkey Anti-Rabbit IgG H&L (Alexa Fluor® 594)	Abcam	ab150076
Rabbit c-Fos primary antibody	Sigma	SAB2100833
Goat anti-CTb primary antibody	Sigma	C730
Donkey anti-goat Alexa Fluor 488	Sigma	SAB4600387
Goat anti-rabbit Alexa Fluor 647	Sigma	AF647
GluA1	Immunoway	YT1923
GluA2	Immunoway	YT1921
GAPDH	Zcibio	ZC-12001
HRP-goat anti-rabbit	Zcibio	ZC-G2106
HRP-goat anti-mouse	Zcibio	ZC-G2104
For optogenetic stimulation and chemogenetical experiment		
rAAV-Ef1α-DIO-hChR2-(H134R)-EYFP-WPRE-pA	Brain VTA	N/A
rAAV-Ef1α-DIO-eNpHR3.0-EYFP-WPRE-pA	OBiO Technology	N/A
rAAV-hEf1α-DIO-EYFP-WPRE-pA	Taitool Bioscience	N/A
AAV2/1-hSyn-Cre	Taitool Bioscience	N/A
rAAV-EF1a-DIO-mcherry-WPRE-pA	Taitool Bioscience	N/A
rAAV-hEF1a- DIO-EYFP-WPRE-pA	Taitool Bioscience	N/A
rAAV-Ef1α-DIO-hChR2-(H134R)-EYFP -WPRE-pA	Taitool Bioscience	N/A
rAAV-hEf1α-DIO-EYFP-WPRE-pA	Taitool Bioscience	N/A
rAAV-EF1a-DIO-hM4D(Gi)-EGFP- WPREs	Brain VTA	N/A
rAAV-EF1a-DIO-EGFP-WPRE-hGH polyA	Brain VTA	N/A
rAAV-CaMKlla-CRE-WPRE-hGH PA	Brain VTA	N/A
Clozapine Noxide （CNO)	Brain VTA	N/A
M/B	Tocris Bioscience	N/A
NBQX	Tocris Bioscience	N/A
Other Chemicals		
Monosodium iodoacetate	Sigma	I2512
Antifade Mounting Medium for Fluorescence	Biosharp	BL701A
PMSF complete protease inhibitors	Thermo Fisher Scientific Inc	36978
SDS	Zcibio	ZC-A0411
Baclofen	Med Chem Express	HYB0007

Muscimol	Med Chem Express	HY-N2313
CTB	Sigma	C9903
Vetbond Adhesive	3M	1469SB
Iisoflurane	RWD Life Science	N/A
Pentobarbital	Shanghai Boding Biotechnology	N/A
Paraformaldehyde	Sinopharm Group Chemical reagent	80096618
Sucrose	Sinopharm Group Chemical reagent	10021418
BSA	Solarbio	SW3015
PBS	Biosharp	BL601A
lidocaine	ChemicalBook	L0156
Experimental models: Organisms/strains		
Rat：Sprague-Dawley	Shanghai Laboratory Animal Center at Chinese Academy of Science	
Mouse：C57BL/6J	SLAC laboratory	
Mouse：CamKⅡα-Cre	Zhang Yuqiu's lab at Fudan University	
Software and algorithms		
Offline Sorter v2.8	Cerebus,Blackrock Neurotech	http://www.creation-tech.com.cn
ANY-maze video tracking software	Stoelting	https://stoeltingco.com
GraphPad Prism v8.0	GraphPad Software	http://www.graphpad.com
MATLAB (2017b)	MathWorks	https://ww2.mathworks.cn
ImageJ	National Institutes of Health	https://imagej.net
NeuroExplorer v5.3	NeuroExplorer	https://www.neuroexplorer.com
Video-tracking system	Shanghai XinRuan Information Technology	https://www.shxinruan.com
Instruments and other materials		
Acupoint nerve stimulator （Model G-6805-2）	Shanghai Medical Electronic Apparatus	N/A
IITC Model 390 Paw Stimulator	IITC Life Science	N/A
Animal behavior tracking device（JLBehv CCD）	Shanghai Jiliang Technology	N/A
EPM apparatus	Shanghai Jiliang Technology	N/A
OFT apparatus	Shanghai Jiliang Technology	N/A
Stereotaxic instruments	Stoelting	N/A
Doric system	Doric Lenses	N/A
Optic fiber	Shanghai June Biotechnology	N/A
Stainless steel needles	Suzhou Yuwell group	N/A
Von Frey filaments	RWD Life Science	N/A
Freezing Microtome	Slee	N/A
Leica Laser Scanning Confocal Microscope	Leica	N/A
Doric system	Doric Lenses	N/A

steel electrodes	Jianyuan Technology	N/A
16 stainless steel microwires	Top-bright	N/A
isolator	A-M Systems	Model 3820

CLINICAL OBSERVATION

Participants and protocol

The study protocol was approved by the ethical committee of the Center of Zhejiang Integrated Traditional and Western Medicine Hospital (ITWMH, No. 202101). The present study was registered on www.chictr.org.cn (Identifier: ChiCTR1800020029). All participants gave written informed consent before the study procedures. The study was in accordance with the Declaration of Helsinki. The data on chronic low back pain (cLBP) were present here.

Patients with cLBP were recruited according to the following criteria.

The inclusion criteria:
(1) Participants of any gender aged 18 to 70 years.
(2) Naive to electroacupuncture (EA) treatment before present study.
(3) Able to understand and complete the consent form and clinical assessment questionnaires without assistance.
(4) Have had cLBP for at least 6 months. Definition of cLBP (a) Chronicity: "defined as a back pain problem that has persisted at least 3 months, and has resulted in pain on at least half the days in the past 6 months." (b) Location: "between the lower posterior margin of the rib cage and the horizontal gluteal fold."
(5) An initial self-reported pain intensity evaluated using the visual analog scale (VAS, 0–10) of at least 4 points (considering that the statistical problem of showing improvement when pain is low on entry, or the target population for a new drug is limited to those with moderate to severe chronic pain [1].

The exclusion criteria:
(1) Having taken any medication or undergone physical therapy in the past week.
(2) Any other serious illnesses or neuropsychiatric diseases.
(3) A history of sleep deprivation or women who were experiencing their menstrual period.
(4) Having consumed coffee or alcohol in the 10 h prior to the EA treatment.

Supervision and training

The study adhered to the Standards for Reporting Interventions in Clinical Trials of Acupuncture (STRICTA) [2] and was supervised by the Data and Safety Monitoring Board of ITWMH. To efficiently conduct clinical experiment and facilitate data replication, all researchers completed human subjects training, including study design, patient registration, basic inclusion and exclusion criteria, group assignment, blinding, assessments, and patient confidentiality.

Participant characteristics

After the screening of 91 patients with cLBP, 51 patients were excluded (13 patients did not completed assessments; 23 patients did not meet inclusion criteria; 13 patients lacked interest in participation; 2 patients feared acupuncture). 40 subjects were recruited and completed all assessments (None of them was familiar with pain research and hypothesis

under study). Given that we focused on the immediate effect of EA on affective and motivation response after pain relief, additional 20 healthy volunteers with matched demographic characteristics were recruited as control. They had no history of neurological or psychiatric disease or chronic pain.
Basic demographic and clinical characteristics of the 40 patients with cLBP and 20 healthy volunteers present in Table S1.

Interventions
EA treatment was conducted by two acupuncturists licensed with at least 10 years of clinical experience. They all completed human subjects training for conducting human subjects study.

<u>EA protocol in patients:</u>
Sterile and single-use acupuncture needles (manufactured by Suzhou Medical Appliance Factory, Shuzhou, China) with a length of 25 to 40mm and a diameter of 0.25 mm were used in the treatment. Six acupuncture points, BL23, BL25, DU3, BL40, DU4, and BL17 point (Table S2), were used for low back pain. Acupuncture points selected in present study were based on some systematic reviews and meta-analysis [3-5], consensus meetings with clinical experts, and our clinical experience. Needles were inserted at traditional depths and angles. The Deqi sensation (a needle-manipulation sensation that indicates effective needling, including numbness, heaviness, soreness, distention, or radiating) was elicited by acupuncture at each acupoint. The acupoint nerve stimulator (Model G-6805-2, Shanghai Medical Electronic Apparatus, China) was used after needle-manipulation. The frequency of stimulation used was 2 Hz. The intensity of the stimulation was varied from 1.0 to 2.0 mA until the patients felt comfortable. The needles were retained at acupuncture points for 30 minutes.

<u>EA protocol in healthy subjects</u>
Healthy subjects experienced the same study procedures as patients did. They received EA at the same six acupuncture points. The electric stimulation and retain of needle in healthy subjects were identical in patients receiving EA.

Procedure and Assessments
The enrolled subjects were guided by investigators independent from EA treatment to finish the assessment interview before or immediately after the EA treatment. A battery of self-report questionnaire was used in the current study. Demographic variables included age, gender, ethnic background, and pain duration.

Similar to our previous study [6], participants were brought into the laboratory office to acclimatize them to specific aspects of the study procedures (including the subjective rating forms and training in relaxation on a day before cue-reactivity test).

Stimuli
We selected ten EA–related slides depicting EA-related objects and people receiving EA were used. Ten neutral slides without EA-related cues served as control stimuli. The EA-relevant slides were generated in our laboratory, prerated by 20 patients with chronic pain, and found to create the expected motivational effects. The no-EA-relevant slides matched the EA-relevant slides with similar scene but without EA cue. Each

no-EA-relevant picture was tested valence score in 30 university students, reflecting that students have neither positive nor negative responses to it. The valence ratings of these pictures (on a scale from 1 (sad) to 10 (happy) ranged from 3.25 to 5.65 with an average rating of 4.35 (SD = 1.25).

Procedure

The study was conducted between 9:00 a.m. and 12:00 p.m. Patients and healthy volunteers received EA stimulation. After EA treatment, participants completed a cue-reactivity procedure that involved presentations of two categories of slides (EA related and no-EA-relevant).

All studies were conducted between 9:00 a.m. and 12:00 p.m. The slides were presented in a fixed semi-random order that was designed to reduce the predictability of slide type. Two additional slides presented at the beginning of the session to orient the subject to the slide presentation were not included in the subsequent analyses. The slides were presented for 8 s each.

Measures

Similar to our previous study [6], the participants provided ratings for each slide in response to the following question when the slide viewing was complete: How much did you want to receive EA treatment? The subjects were instructed to respond on an 11-degree numerical rate scale (NRS), with “0” indicating not at all and “10” indicating extremely.

In addition, a battery of self-report questionnaire included age, gender, ethnic background, and pain duration were collected. The self-reported pain and expectation of pain relief of all individuals were assessed using the visual analogue scale (VAS), a 10 cm horizontal line with a 0 at the start representing no pain/EA expectation and a 10 at the end representing extreme pain/EA expectation. Furthermore, the Positive and Negative Affect Scale (PANAS) was used to assess affective states before and after EA stimulation. The PANAS scale is a well-validated measure of subjective affective state, comprising of 20 items on positive affect (PA) and negative affect (NA). The PANAS scale is broken into two sections to measure both the PA and NA. PA refers to the tendency to experience positive emotions, and the higher the positive affect the more prevalent these emotions are. NA is the opposite and refers to the level of negative emotions. It is considered an effective screening measure for PA and NA and has been widely used in China [7, 8].

Statistical Analysis

The raw data were initially imported into EXCEL sheets and checked manually. Only the data of subjects who accomplished the whole assessment procedures were included into analysis. The statistical tests were performed using GraphPad Prism software (version 8). Before applying ANOVA or the t-test, descriptive statistics were performed on all data. The Z-scores all fell within ±1.96, indicating that the data follows a normal distribution. Baseline demographic variables and clinical characteristics were compared across groups using one-way analyses of variance (ANOVAs) or Pearson χ^2 testes for categorical variables. Two-way analysis of variance (ANOVA) with between-subject factor group (EA, Sham EA) and within-subject factor condition (before and after treatment) was used

to analyze pain scales, PA and NA scores. Mean levels of motivational scales were analyzed using a two-way analysis of variance (ANOVA) with between-subject factor group (patients and healthy controls) and within-subject factor condition (category: neutral, EA-related). Following the initial analysis, separate analyses were performed to determine each measure separately with between-subject factor and group. A series of Pearson product-moment correlations also were performed to assess the associations between the pain relief and emotional measures. The accepted level of statistical significance was a threshold of $P < 0.05$.

Reference

1. M. C. Rowbotham, What is a "clinically meaningful" reduction in pain? Pain 94, 131-132 (2001).
2. H. MacPherson, A. White, M. Cummings, K. Jobst, K. Rose, R. Niemtzow, Standards for reporting interventions in controlled trials of acupuncture: the STRICTA recommendations. Complement. Ther. Med. 9, 246-249 (2001).
3. J. Mu, A. D. Furlan, W. Y. Lam, M. Y. Hsu, Z. Ning, L. Lao, Acupuncture for chronic nonspecific low back pain. Cochrane. Database. Syst. Rev. 12, CD013814 (2020).
4. B. M. Berman, H. M. Langevin, C. M. Witt, R. Dubner, Acupuncture for chronic low back pain. N. Engl. J. Med. 363, 454-461 (2010).
5. J.C. Deare, Z. Zheng, C. C. Xue, J. P. Liu, J. Shang, S. W. Scott, G. Littlejohn, Acupuncture for treating fibromyalgia. Cochrane. Database. Syst. Rev. 2013, CD007070 (2013).
6. S. Liu, W. Zhou, J. Zhang, Q. Wang, J. Xu, D. Gui, Differences in cigarette smoking behaviors among heroin inhalers versus heroin injectors. Nicotine. Tob. Res. 13, 1023-1028 (2011).
7. M. Kim, Z. Wang, Factor structure of the PANAS with bayesian structural equation modeling in a Chinese sample. Eval. Health. Prof. 45, 157-167 (2022).
8. J. D. Liu, R. H. You, H. Liu, P. K. Chung, Chinese version of the international positive and negative affect schedule short form: factor structure and measurement invariance. *Health. Qual. Life. Outcomes.* **18**, 285 (2020).

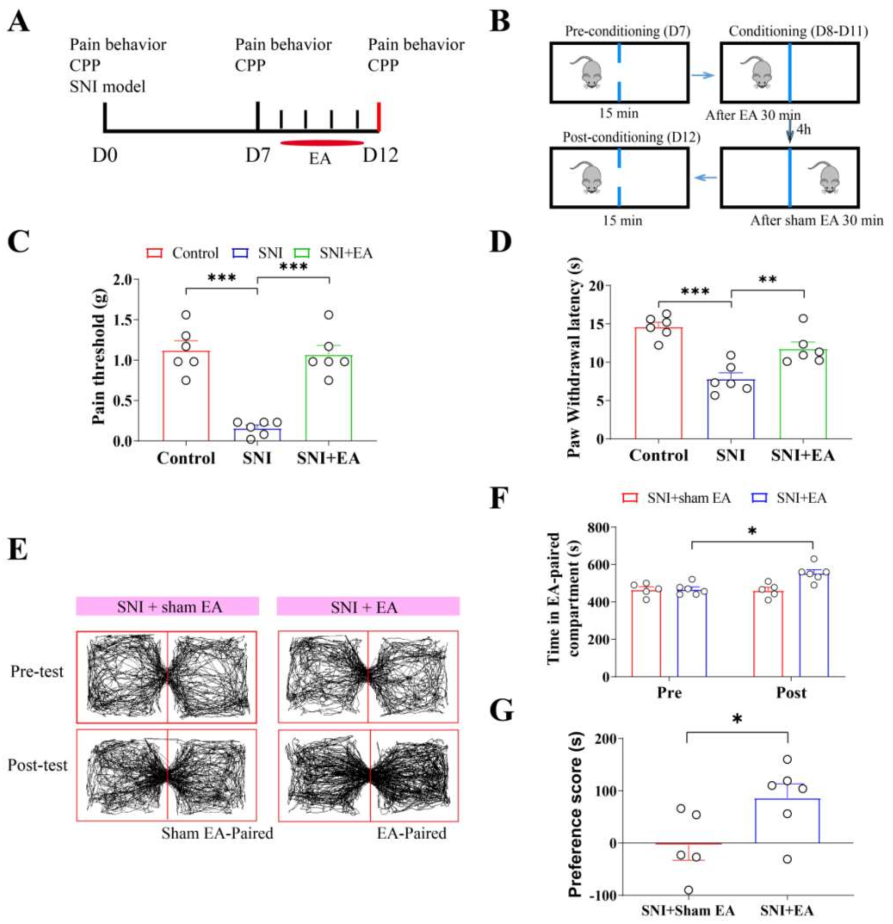

Fig. S1 EA induces analgesia and CPP behavior in SNI mice. (A) Schematic showing the protocol of experiment. (B) Procedure to elicit and test EA induced-CPP behavior. (C) Pain threshold ($F_{(2, 15)}$ = 32.09, P<0.0001). (D) Paw withdrawal latency (s) ($F_{(2, 15)}$ = 20.54, P<0.0001). (E) The trajectory of SNI mice in a CPP apparatus during pre-test phase and post-test phase. (F) Time in EA-paired compartment (s) before and after EA ($F_{(1, 9)}$ = 5.189, P=0.049, Two-way ANOVA with Sidak's multiple comparisons test, n = 5-6 mice/group). (G) CPP scores. Two-tailed unpaired t-test. t=3.495, P=0.007. All data were presented as mean ± S.E.M. One-way ANOVA with Sidak's multiple comparisons test (C, D). n = 6 mice/group. *P< 0.05, **P< 0.01, ***P< 0.001.

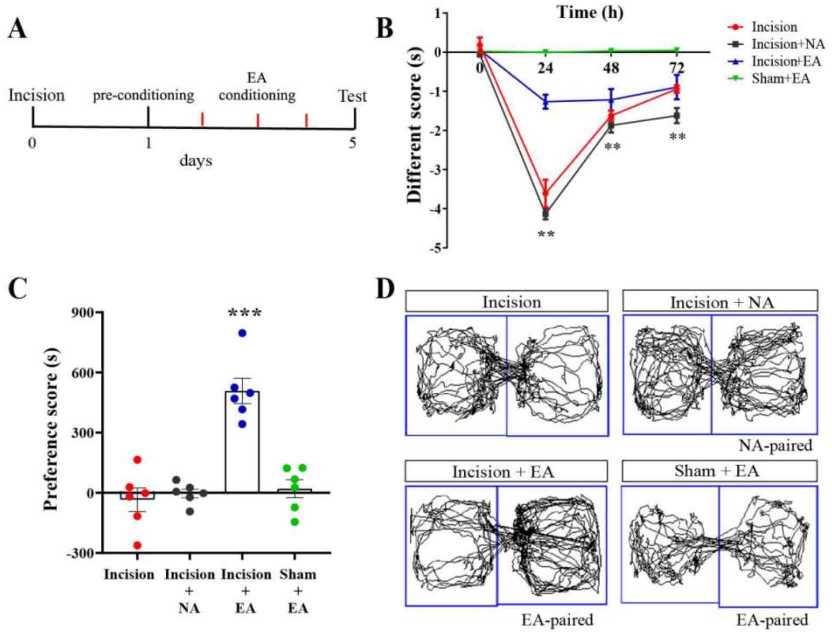

Fig. S2 EA induces analgesia and CPP behavior in incisional injury pain (INP) rats. (A) Schematic showing the protocol of experiment. (B) EA attenuated pain hypersensitivity in INP rats ($F_{(3, 20)}$ = 26.97, P<0.0001, Two-way ANOVA with Sidak's multiple comparisons test, n = 6 rats/group). Different scores, which was calculated by subtracting the PWL of the untreated paw from the PWL of the injured paw. **P< 0.01 versus Incision, EA at non-acupoints (Incision + NA), and EA in sham operated animals (Sham + EA). (C) Preference scores ($F_{(3, 20)}$ = 26.78, P<0.0001, one-way ANOVA with Sidak's multiple comparisons test, n = 6 rats/group). ***P< 0.001 vs. Incision, Incision+ NA, and Sham + EA. (D) Real-time movement traces among the groups during CPP test. Data are shown as mean ± S.E.M.

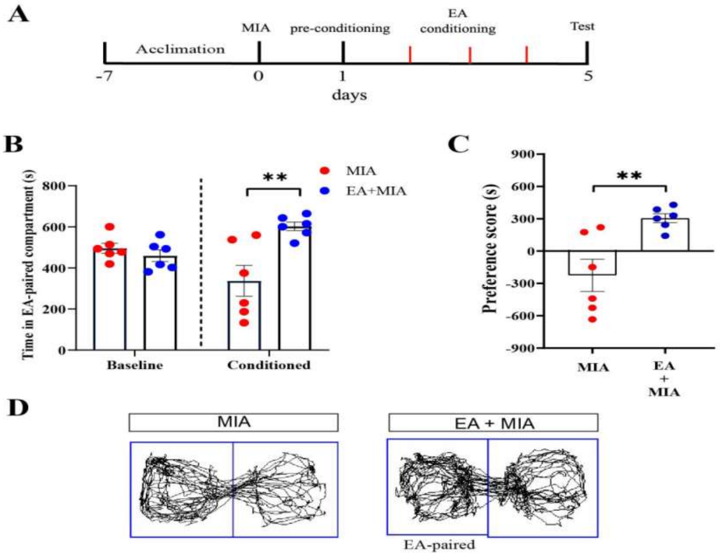

Fig. S3 EA induces a significant preference for EA-paired compartment in MIA rats. (A) A timeline of MIA modeling, EA-induced CPP, and CPP test. (B) Time in EA-paired compartment (s) before and after EA ($F_{(1, 10)}$ = 14.06, P<0.0001, Two-way ANOVA with Sidak's multiple comparisons test, n = 6 mice/group). (C) CPP scores. Two-tailed unpaired t-test. t=3.422, P=0.007. (D) Representative real-time place preference tracks between the groups during CPP test. Data are shown as mean ± S.E.M. **P< 0.01.

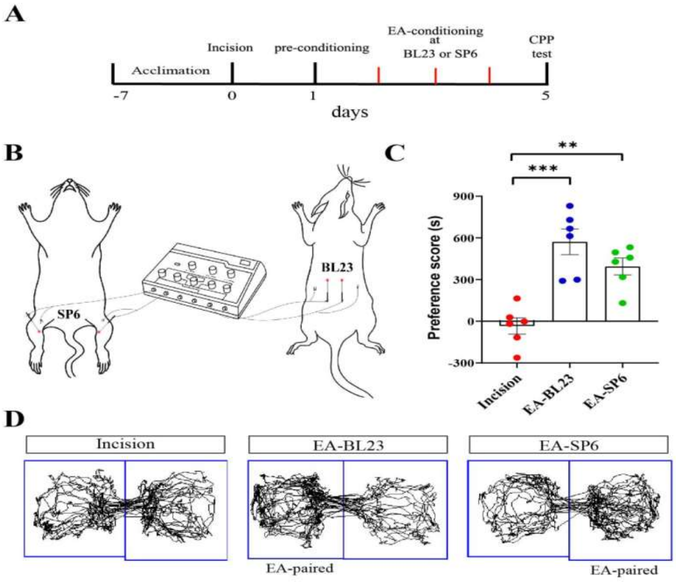

Fig. S4 EA at BL.23 or SP.6 acupuncture points produces a significant EA-related preference in INP rats. (A) A timeline of INP modeling, EA-induced CPP, and CPP test. (B) Schematic representation of placement of EA at BL23 or SP6 acupuncture points. (C) Preference scores ($F_{(2, 15)} = 18.65$, $P<0.0001$, one-way ANOVA with Sidak's multiple comparisons test, n = 6 rats/group). (D) Representative real-time place preference tracks among the groups during CPP test. Data are shown as mean ± S.E.M. **P< 0.01, ***P< 0.001.

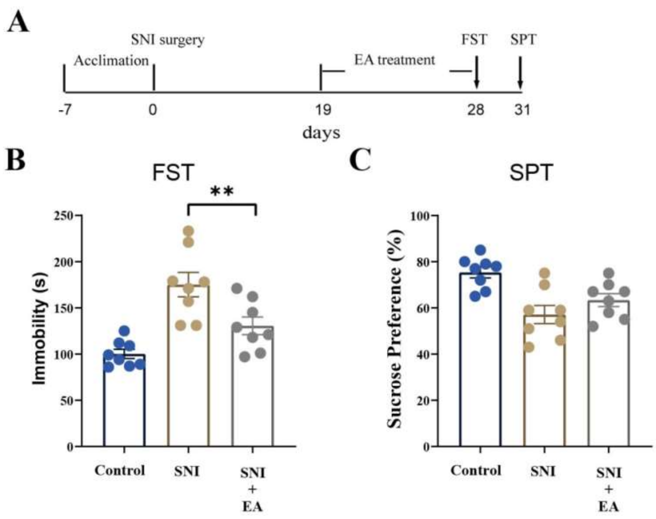

Fig. S5 Effects of EA on forced swimming (FST) and sucrose preference (SPT) in SNI rats. (A) Schedule of EA on FST and SPT tests in SNI rats. From day − 7 to day 0, rats were acclimated to new environment. Rats were conducted SNI surgery on day 0. Rats were received EA treatment once a day from day 19 to day 28 after SNI. FST and SPT were performed on day 28 and 31, respectively. (B) EA significantly shortened the immobile time of SNI rats in FST ($F_{(2, 21)}$ = 14.78, P<0.0001). (C) The effect of EA on sucrose preference in SNI rats. There was a trend for EA to increased sucrose intake, although this effect did not achieve statistical significance (P = 0.16). All data are expressed as the mean ± S.E.M. One-way ANOVA with Sidak's multiple comparisons test, n = 8 rats/group; **P< 0.01.

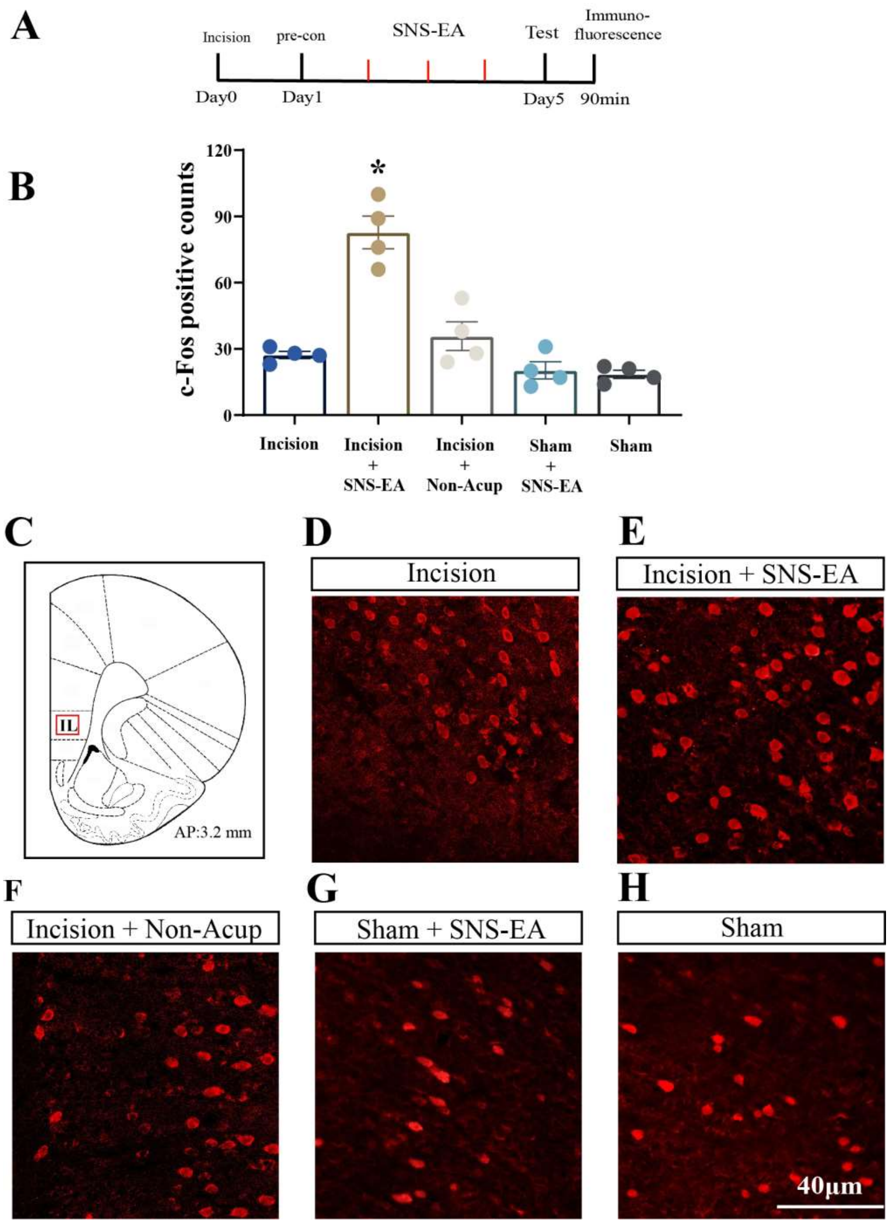

Fig. S6 EA-induced CPP exhibits a significant increase in numbers of c-Fos+ neurons in the IL. (A) Schematic showing the protocol of experiment. (B) Quantitative analysis of c-Fos+ neurons in the IL following EA-induced CPP test ($F_{(4, 15)}$ = 29.79, P<0.0001, one-way ANOVA with Sidak's multiple comparisons test, n = 4 rats/group). ***P< 0.001 vs. Incision, EA at non-acupoints (Incision+NA), sham operated (Sham) and sham operated (Sham) + EA group. Data are expressed as the mean ± S.E.M. (C) Schematic coronal section through the IL illustrated placement of the rectangle in the IL where c-Fos+ neurons were counted. (D)-(H) Representative coronal sections showing Fos immunoreactivity in the IL in different groups. Scale bar, 40 μm.

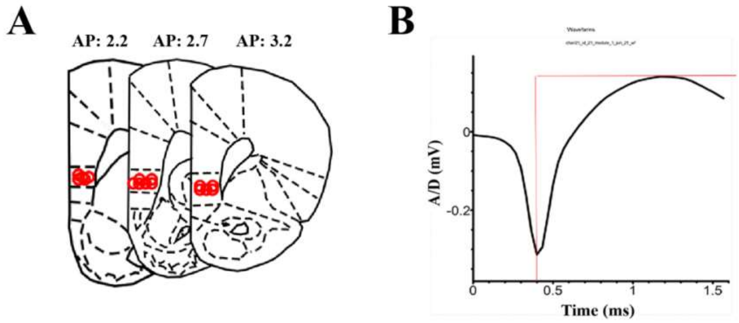

Fig. S7 Placement of recording wire tips and characterization of waveform in extracellular recordings in the IL. Related to Fig. 3. (A) Schematics illustrating the placement of recording wire tips for IL-implanted animals. (B) Representative waveform used for analysis of action potential width of putative glutamatergic pyramidal cells. Width measurements were taken as the time between spike valley and the following peak.

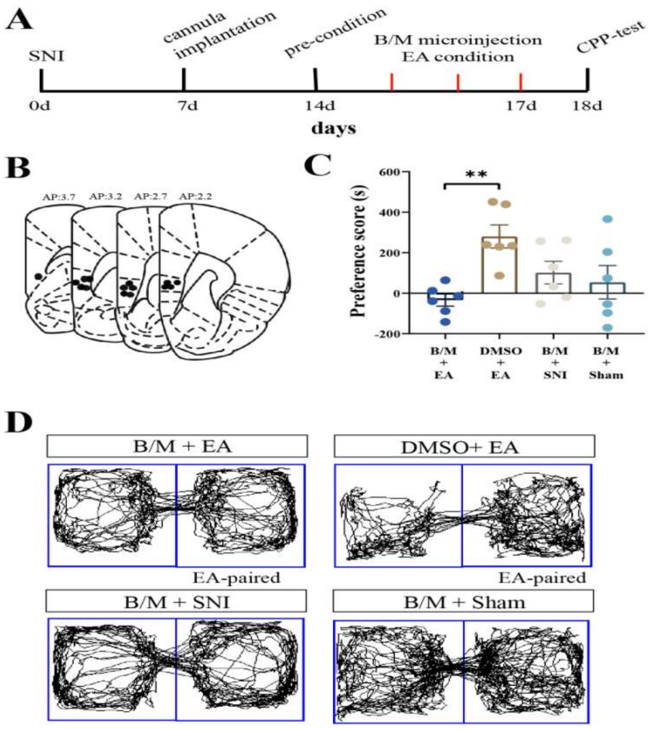

Fig. S8 Transient IL inactivation disrupts the effects of EA on CPP behaviors in SNI model rats. (A) A timeline of SNI modeling, EA treatment, stereotactic injection, and behavioral testing protocols. (B) Microinfusion cannula placements. The numbers indicate distance from Bregma in millimeters. (C) The effect of transient IL inactivation on EA preference in SNI rats. $F_{(3, 20)} = 4.966$, P<0.001, one-way ANOVA with Sidak's multiple comparisons test, n = 6 rats/group. Data are expressed as the mean ± S.E.M. **P< 0.01. (D) Real-time movement traces during CPP test in different groups.

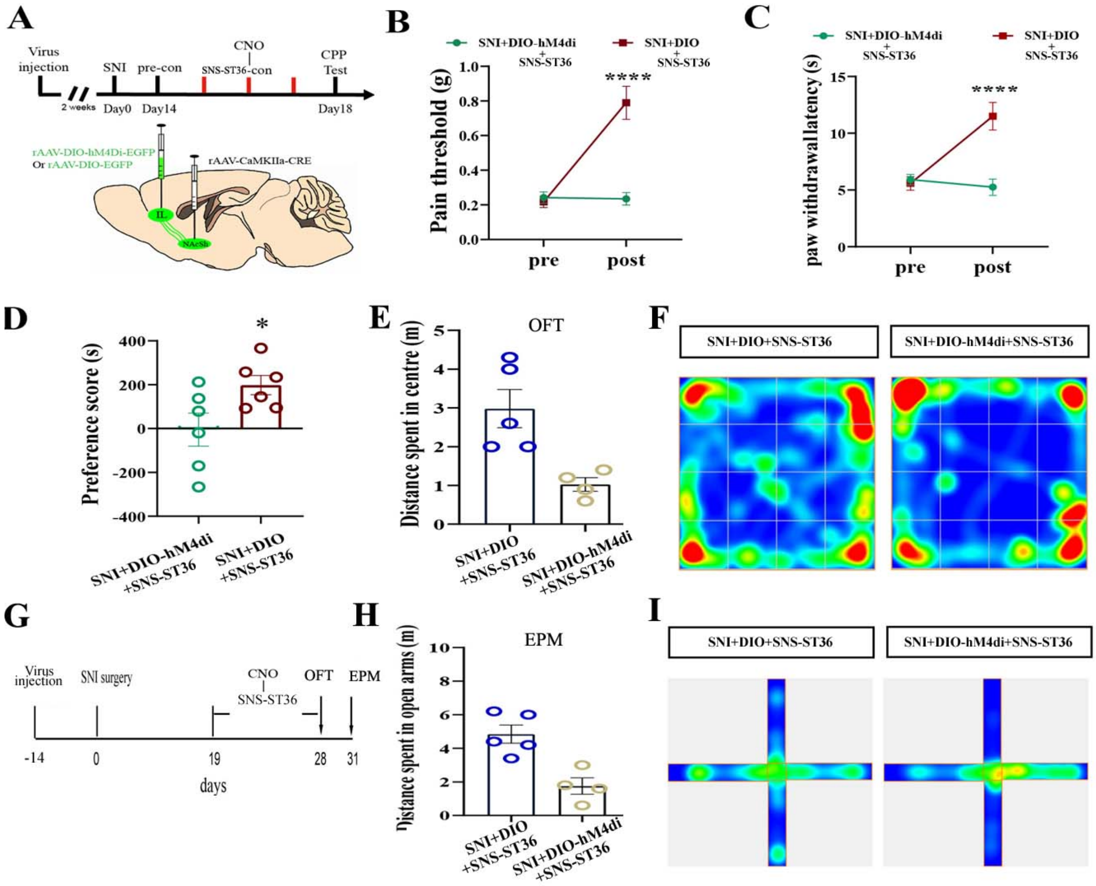

Fig. S9 Inactivating IL^{Glu} – NAc shell pathway reserves EA-induced analgesia and affective-motivational behaviors. (A) Schematic diagram showing the timeline of experiment and bilateral injection rAAV-EF1a-DIO-hM4D(Gi)-EGFP-WPREs or rAAV-EF1a-DIO-EGFP -WPRE-hGH polyA into the IL and rAAV-CaMKlla-CRE-WPRE-hGH PA into the NAc shell. Only mice with virus expression confined to IL were included in the study. In our pilot study, CNO injected 30 min before EA significantly reduced IL c-Fos expression induced by EA stimulation in DREADD-infected neurons (Data not shown). (B)-(D) The increased thresholds of mechanical allodynia and thermal hyperalgesia and CPP scores induced by EA were inhibited by clozapine-N-oxide (CNO) intraperitoneal injection. (B) Pain threshold ($F_{(1, 10)}$ = 23.05, P=0.0007). (C) Paw withdrawal latency ($F_{(1, 10)}$ = 8.234, P=0.0167). Two-way ANOVA with Sidak's multiple comparisons test, n = 6 rats/group. (D) CPP scores (Two-tailed unpaired t-test. t=3.682, P= 0.0143). (E) Distance in the central area during OFT (Two-tailed unpaired t-test. t=3.376, P=0.012). (F) Heat maps for behavior tracking of rats during OFT. (G) Schematic diagram showing the timeline of experiments for OFT and EPM. (H) Distance in the central area during EPM (Two-tailed unpaired t-test. t=4.112, P=0.005). (I) Heat maps for behavior tracking of rats. All data were presented as mean ± S.E.M. *P< 0.05, ****P< 0.0001.

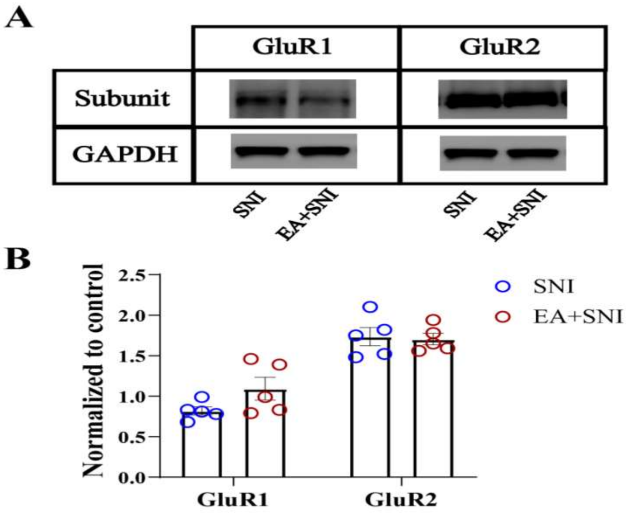

Fig. S10 EA does not cause changes in levels of GluR1 or GluR2 subunits in the synaptoneurosome fractions of NAc shell in SNI rats. (A) Representative Western blot results for GluR1 or GluR2 subunits after treatment with or without EA. (B) The protein expression of GluR1 (t=1.823, P=0.396) or GluR2 subunits (t=2.227, P=0.341) in the NAc shell were measured after EA treatment in SNI rats. Two-tailed unpaired t-test. All data were presented as mean ± S.E.M.

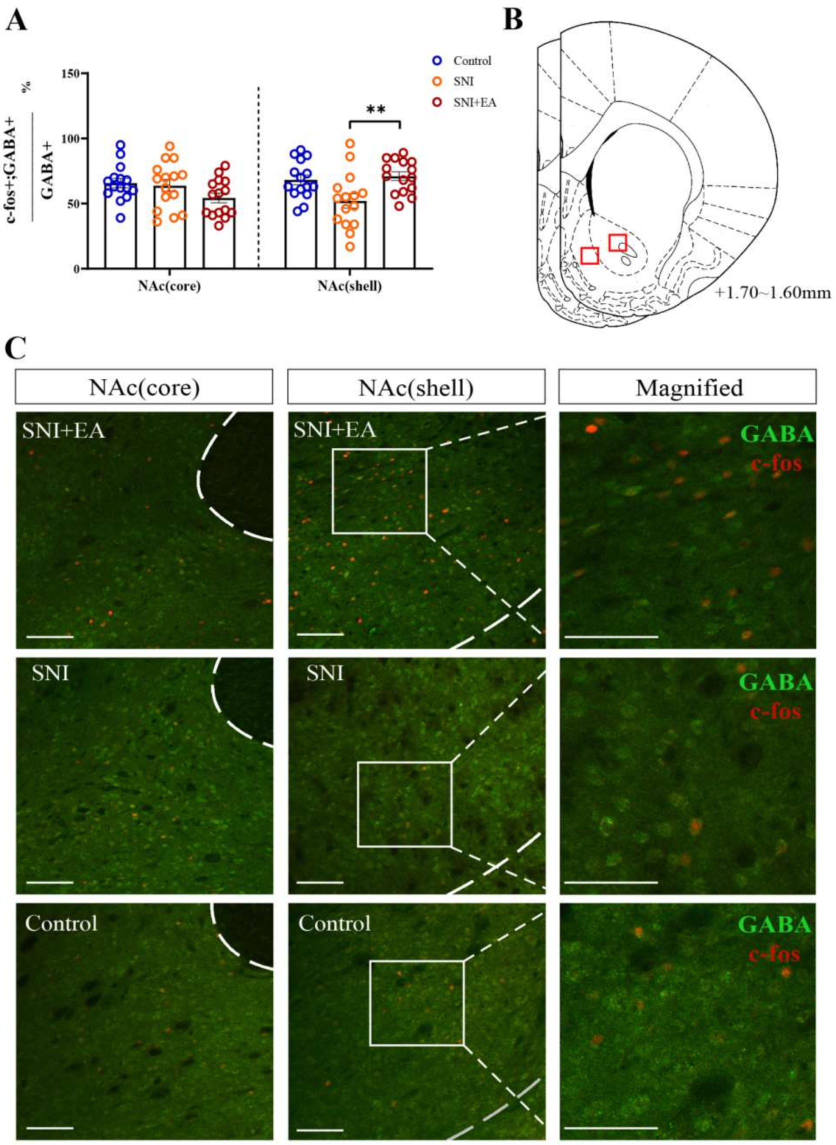

Fig. S11 EA activates Fos+ GABA neurons in the NAc shell but not in the NAc core in SNI rats. (A) Quantitative analysis of c-Fos+/GABA+ neurons colocalization in the NAc shell ($F_{(2, 42)} = 5.688$, P=0.007) and core ($F_{(2, 42)} = 2.310$, P=0.112). One-way ANOVA with Sidak's multiple comparisons test, n =15 rats/group. Data are expressed as the mean ± S.E.M. **P< 0.01. (B) Schematic coronal section through the NAc where double-labelled cells were counted. (C) Representative photomicrograph showing c-Fos+/GABA+ neurons colocalization in the NAc in different groups. Scale bar, 100μm; 40μm (magnified).

Table S1. Basic demographic and clinical Characteristics of included participants

	Group	
	patients	Healthy Control
Sample size (N)	40	20
Gender (F/M)	26/14	12/8
Age (years)	41.7±12.5	38.7±8.4
Ethnicity (%)		
Han	38 (95)	19 (95)
Minority	2 (5)	1 (5)
Pain duration (Month,SD)	43.0±6.9	—

Table S2. Location of acupuncture points selected.

Point Name	Location
BL 17	At the level of the lower border of the spinous process of the seventh thoracic vertebra, 5cm lateral to the posterior midline.
BL 23	At the level of the lower border of the spinous process of the second lumbar vertebra, 5cm lateral to the posterior midline.
BL 25	At the level of the lower border of the spinous process of the fourth lumbar vertebra, 5cm lateral to the posterior midline.
BL 40	Midpoint of the transverse crease of the popliteal fossa, between the tendons of the biceps femoris and semitendinosus muscles.
GV 3	Below the spinous process of the fourth lumbar vertebra, at the level with the iliac crest.
GV 4	Below the spinous process of the second lumbar vertebra, on the posterior median line.
